# Cell‐specific protein expression in Alzheimer's disease prefrontal cortex

**DOI:** 10.1002/alz.70339

**Published:** 2025-06-04

**Authors:** Maryam Gholampour, Malay K. Basu, Russell H. Swerdlow, Xinming Zhuo, Mohammad Haeri

**Affiliations:** ^1^ Department of Pathology & Laboratory Medicine University of Kansas Medical Center Kansas City Kansas USA; ^2^ Alzheimer's Disease Research Center University of Kansas Kansas City USA; ^3^ Department of Neurology University of Kansas Medical Center Kansas City Kansas USA; ^4^ Department of Pathology and Cell Biology Columbia University Irving Medical Center New York New York USA

**Keywords:** Alzheimer's disease, brain cell types, GeoMx DSP, neprilysin, spatial proteomics

## Abstract

**INTRODUCTION:**

Analyzing the proteomes of different brain cell types is fundamental for understanding the pathophysiology of Alzheimer's disease (AD). However, spatial analysis of these diverse and limited cell populations poses significant challenges.

**METHODS:**

The GeoMx Digital Spatial Profiler (DSP) platform was used to analyze protein level in the prefrontal cortex of AD and non‐AD brains. The platform interrogated 76 proteins and used immunofluorescence to distinguish between three cell types.

**RESULTS:**

Neprilysin (NEP), which promotes amyloid beta degradation, was significantly higher in AD neurons and microglia. Lysosome‐associated membrane protein 2A (LAMP2A) level was higher in neurons of individuals with AD compared to a control group. In addition, markers of neuroinflammation, such as CD11c, CD11b, and CD163, were also elevated in AD neurons.

**DISCUSSION:**

Our findings indicate the DSP platform effectively facilitates cell‐specific snapshots of the AD brain proteome.

**Highlights:**

- The expression of 76 proteins was studied in neurons, astrocytes, and microglia.
- We identified 18 differentially expressed proteins in AD neurons.
- Neprilysin was upregulated in neurons and microglia.

## 1 BACKGROUND

Alzheimer's disease (AD) is a complex neurodegenerative disorder with multifactorial etiology. Although genetic, environmental, and lifestyle factors are known to influence the onset of AD, none has been definitively proven as the cause of late‐onset AD. Postmortem diagnosis of AD relies on the presence of specific neuropathological hallmarks: amyloid plaques and neurofibrillary tangles (NFTs). The formation of these aggregates correlates with neuroinflammation, synaptic dysfunction, neurodegeneration, and ultimately, the cognitive decline that defines clinical AD. Imaging and biomarker studies suggest an extended preclinical phase, with AD pathology developing decades before dementia manifests.^1^, ^2^

Despite significant research efforts, the underlying causes and progression of AD remain poorly understood, hindering the development of effective treatments. This has led to numerous negative outcomes from clinical trials^3^ and preventive measures such as vaccines.^4^ Traditional hypothesis‐driven research focused on specific proteins has yielded valuable insights identifying amyloid beta (Aβ) as the primary component of plaques,^5^ tau protein as the core constituent of NFTs,^6^ and apolipoprotein E (*APOE*) as the most significant genetic risk factor for late‐onset AD,^7^, ^8^ but it has limitations in comprehensively understanding the complex AD proteome. To overcome these challenges, omics technologies offer a promising approach to identify novel disease‐associated genes and proteins, providing a comprehensive view of AD pathogenesis.^9^, ^10^, ^11^ Although genomics and epigenetics have contributed to identifying genetic risk factors, proteomics holds the potential to reveal dynamic protein changes associated with disease progression.^12^ A key challenge in these analyses lies in achieving a balance between sensitivity, specificity, and multiplexity. Increased complexity and precision often come at the cost of sensitivity, thereby limiting the detection of low‐abundance analytes. Mass spectrometry has emerged as a promising tool for enhancing sensitivity and multiplexity.^13^, ^14^ However, a major limitation of mass spectrometry for multiplexed proteomic analysis is the irreversible destruction of the sample, preventing further analysis.^15^ This becomes particularly important in spatial omics studies that aim to integrate transcriptomic and proteomic data while preserving spatial information.^16^

Spatial proteomics integrates data on protein expression within cells with their precise localization within a tissue.^17^ This spatial information can be crucial for understanding the differential vulnerability of cells and the precise mechanisms underlying the propagation of pathology within the brain, especially in the context of neurodegenerative diseases potentially exhibiting prion‐like transmission.^18^, ^19^ Additional spatial information relevant to neurons and other cell types includes proximity to vessels, different layers of the cortex, depth of the sulci, or curve of the gyri, among others. Many studies have shown that significant biological changes relevant to AD pathophysiology may not be reflected by changes in messenger RNA (mRNA) abundance or co‐expression.^12^, ^20^, ^21^ This highlights the importance of integrating data across different omics levels to gain a deeper understanding of the disease and identify potential therapeutic targets.

Although significant advancements have been made in commercially available spatial transcriptomics technologies, few platforms currently offer proteomic capabilities. NanoString GeoMx Digital Spatial Profiler (DSP) offers the distinct advantage of simultaneously detecting RNA and protein in a single run, making it a valuable tool for researchers with limited sample availability.^22^ The recently launched CosMx Spatial Molecular Imaging (SMI) technology promises ultra‐high‐resolution imaging of 64 targets alongside four dedicated cell segmentation markers on a single slide. The latter has applications available for mouse neuroscience protein panels, and potentially for humans.^23^ This technology displays single‐cell and subcellular resolution, surpassing the 50 µm resolution of GeoMx DSP.

In this pilot study, we investigated protein expression differences across three cell types within post‐mortem AD brain prefrontal cortices utilizing the GeoMx DSP technology.

## 2 METHODS

### 2.1 Cases

We obtained samples from Brodmann Area 8/9 (as detailed in Table 1) from four AD brains confirmed by histopathological examination.^24^ In addition, we obtained four non‐demented age‐matched control brains from the University of Kansas Alzheimer's Disease Research Center (KU ADRC) Neuropathology Core. To ideally accentuate differences between the groups, we selected AD brains from *APOE* ε4 carriers and non‐AD brains from *APOE* ε4 non‐carriers.

**RESEARCH IN CONTEXT**

1. **Systematic review**: Spatial proteomics offers a powerful approach to investigate the complex cellular and molecular mechanisms underlying Alzheimer's disease (AD). Most studies have relied on mass spectrometry for multiplexed proteomics; however, this technique irreversibly destroys samples, thereby limiting further analysis and preventing the acquisition of spatial data.
2. **Interpretation**: The value of our study lies in investigating protein expression across three different cell types (neurons, astrocytes, and microglia) in the human prefrontal cortex from controls and patients with AD, with special emphasis on AD cases that are apolipoprotein E (*APOE*) ε4 carriers.
3. **Future directions**: Investigating how protein expression varies across different layers of gray matter in the prefrontal cortex of human AD brains is essential.

**TABLE 1:** Demographics and pathologic features of AD and control cases.

Case number	Subgroup	*APOE*	Sex	Age (years)	PMI (hours)	Thal (A)		Braak (B)		CERAD (C)	
1	Control	E3/E3	M	82	22.1	2	A1	2	B1	0	C0
2	Control	E3/E3	M	95	39	0	A0	2	B1	0	C0
3	Control	E3/E3	M	77	4.7	0	A0	3	B2	0	C0
4	Control	E3/E3	F	60	39.6	0	A0	0	B0	0	C0
5	AD	E4/E4	M	76	5.0	5	A3	5	B3	3	C3
6	AD	E4/E4	F	75	17.7	5	A3	6	B3	3	C3
7	AD	E3/E4	F	69	9.4	5	A3	6	B3	3	C3
8	AD	E4/E4	M	70	17.6	5	A3	6	B3	3	C3

Abbreviations: AD, Alzheimer's disease; *APOE*, apolipoprotein E; Braak (B), Braak neurofibrillary tangle stage; CERAD (C), Consortium to Establish a Registry for Alzheimer’s Disease score; PMI, postmortem interval; Thal (A), Thal amyloid phase.

### 2.2 Immunohistochemistry

For immunohistochemistry, formalin‐fixed, paraffin‐embedded (FFPE) histological sections were dewaxed in xylene, rehydrated in an ethanol series (100%, 95%, and 70%), and then rinsed with dH_2_O. Sections were subjected to antigen retrieval in 1X citrate buffer, pH 6.1 (Dako‐S1699) for 10 min at 99°C, rinsed another time with dH_2_O, and blocked with methanol containing 3% H_2_O_2_ treatment for 10 min. Slides were incubated with PHF1‐Tau primary antibody (kind gift from Dr. Peter Davies) at a dilution of 1:1000 overnight at 4°C. The next day, slides were rinsed with phosphate‐buffered saline (PBS) buffer and incubated with ImmPRESS horseradish peroxidase (HRP)–labeled secondary antibody (Vector Laboratories‐MP‐6402‐15) for 30 min at room temperature. Finally, slides were subjected to hematoxylin and eosin (H&E) staining and dehydrated in an ethanol series (70%, 95%, and 100%) and xylene. The protocol was identical for Aβ, except for the antigen retrieval step, which was performed in 99% formic acid for 10 min at room temperature, and the primary antibody used was Dako‐M0872 at a dilution of 1:100.

### 2.3 Spatial proteomics analysis

Upon the completion of deparaffinization, FFPE sections were subjected to treatment with a combination of 76 distinct antibodies (Table S1); each antibody was conjugated to a unique ultraviolet (UV) photocleavable oligonucleotide tag. Specific antibodies labeled with fluorescent markers were used as morphological indicators for designated cells within the regions of interest (ROIs).^25^ Specific cell types were selected using morphology of neurons (neuronal nuclei (NeuN); ABN78, Millipore Sigma, conjugated with Alexa Fluor 647), microglia (Ionized calcium‐binding adapter molecule 1 (Iba1); 48934S, Cell Signaling Technology, conjugated with Alexa Fluor 594), and astrocytes (glial fibrillary acidic protein (GFAP); NBP2‐33184DL594, Novus Biologicals conjugated with Alexa Fluor 488). Nuclei were stained with SYTO 83 (S11364, Thermo Fisher Scientific). ROIs measuring 660 × 785 µm (area: 518100 µm^2^) were targeted within brain gray matter. To isolate protein‐specific oligonucleotides, individual ROIs were exposed to UV light on the GeoMx DSP. The released oligonucleotides were collected on a 96‐well plate and hybridized to optical barcodes. The nCounter platform analyzed these barcoded oligonucleotides, generating spatially resolved protein counts for each ROI. These counts were normalized to ERCC RNA control counts and further normalized using the signal‐to‐noise ratio. Log2‐transformed data from 76 proteins were used for subsequent analysis. Previous studies have shown alterations in internal control proteins such as GAPDH, histone H3, and S6 in AD.^26^, ^27^, ^28^ Therefore, they may not be reliable as quality control markers due to their own disease‐related changes.

### 2.4 Statistics

Expression differences between AD and control cases were assessed using R Studio version 4.1.2. The Benjamini–Hochberg (BH) method and the *t*‐test of means were applied to account for multiple comparisons. Volcano plots were generated using log2‐normalized data of significantly expressed targets (adjusted *p*‐value ≤ 0.05); box plots were created using GraphPad Prism version 9 (GraphPad Software, Inc., La Jolla, CA, USA).

## 3 RESULTS

Prefrontal cortical samples were obtained from four controls and four AD subjects. Case demographics for the groups are provided in Table 1. The mean age at death of the AD cohort was 72.5 years (range: 69–76 years), and the mean age at death of the non‐demented cohort was 78.5 years (range: 60–95 years). The diagnosis of AD was made by standard NACC neuropathological evaluation (https://naccdata.org/data‐collection/forms‐documentation/np‐11).

**FIGURE 1:**
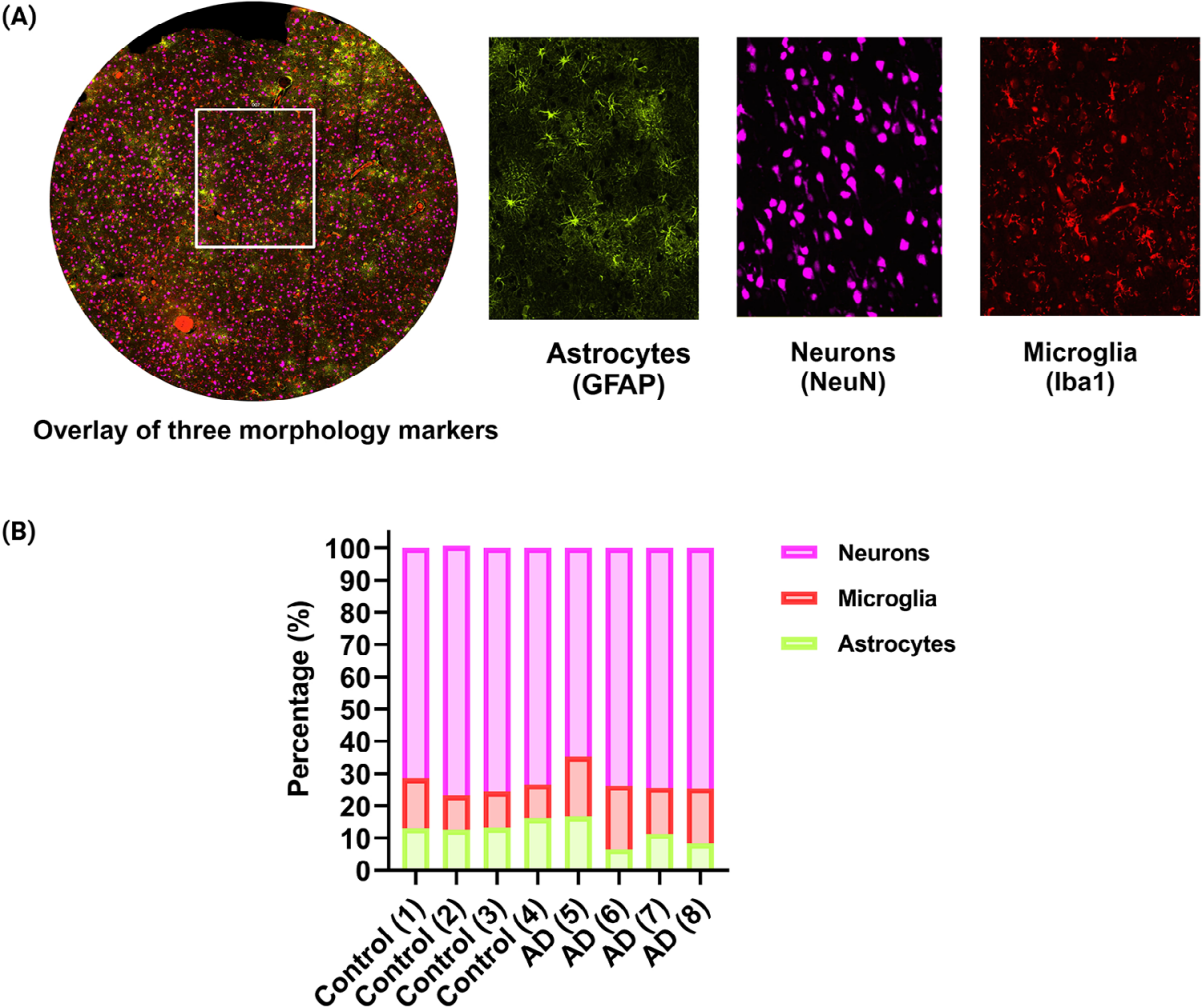
(A) Multicolor immunofluorescence localizes the expression of specific markers GFAP, NeuN, and Iba1 in AD postmortem FFPE tissue. (B) Distribution of cell types in eight ROIs from AD and controls. AD, Alzheimer's disease; FFPE, formalin‐fixed, paraffin‐embedded; GFAP, glial fibrillary acidic protein; Iba1, ionized calcium‐binding adapter molecule 1; NeuN, neuronal nuclei; ROIs, regions of interest.

We used three morphological markers: GFAP for astrocytes, NeuN for neurons, and Iba1 for microglia. Each marker had a specific fluorophore (Figure 1A, Table S2) to allow identification of each cell type through immunofluorescence. Counted cell types as well as the total number of cells in each ROI are summarized in Table 2. As shown in Figure 1B, the average percentage of neurons in the control group was 74.7%, compared to 71.8% in the AD cohort. For microglia, the control group had 11.9%, whereas the AD group had 17.4%. Astrocytes accounted for 13.7% of the total cells in the control group and 10.6% in the AD group.

**TABLE 2 alz70339-tbl-0002:** Counted cell types reported by NanoString GeoMx DSP.

ROI	Subgroup	Nuclei (GFAP)	Nuclei (Iba1)	Nuclei (NeuN)	Total
1	Control	60	72	331	983
2	Control	63	54	389	754
3	Control	60	51	345	667
4	Control	89	57	404	769
5	AD	72	80	280	649
6	AD	35	105	394	953
7	AD	61	78	405	749
8	AD	46	94	410	674

Abbreviations: AD, Alzheimer's disease; DSP, Digital Spatial Profiler; GFAP, glial fibrillary acidic protein; Iba1, ionized calcium‐binding adapter molecule 1; NeuN, neuronal nuclei; ROI, regions of interest.

We employed a human neuroscience panel (Alzheimer's Pathology, Alzheimer's Pathology Extended, Autophagy, Glial Cell Subtyping, Parkinson's Pathology) from NanoString to investigate the expression of 76 different proteins in these cell groups.[Table alz70339-tbl-0002] Proteins studied are classified in 14 classes in Table S3.

**FIGURE 2 alz70339-fig-0002:**
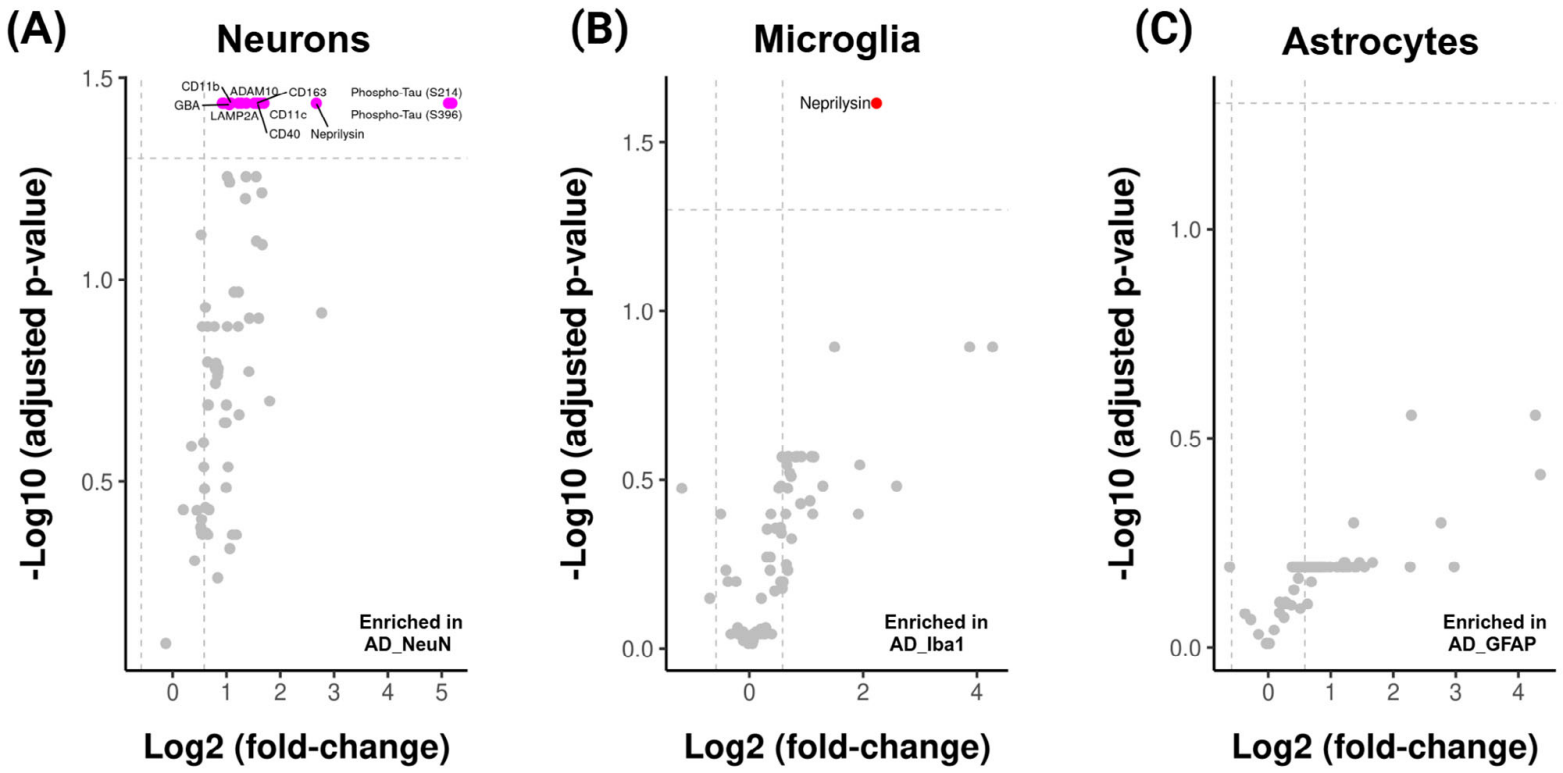
Volcano plot of differential expression of proteins in neurons (A), microglia (B), and astrocytes (C) between the AD and control brains (The threshold criteria for statistical significance, *p*.adj < 0.05 and |FC| > 1.5, were considered significant and are shown as dashed gray lines. Proteins with significant changes are color‐coded: magenta for neurons and red for microglia). AD, Alzheimer's disease; GFAP, glial fibrillary acidic protein; NeuN, neuronal nuclei; Iba1, ionized calcium‐binding adapter molecule 1.

The threshold criteria for statistical significance are *p*.adj < 0.05 and |FC| > 1.5. Of the 76 analyzed proteins, 18 were differentially expressed in the neurons of the AD group compared to the control group. The most significantly upregulated proteins in AD neurons were neprilysin (NEP), CD163, ADAM10, CD11c, CD40, lysosome‐associated membrane protein 2A (LAMP2A) glucocerebrosidase (GBA), and CD11b, and anticipated phosphorylated tau (S214 and S396) (labeled in Figure 2A.)

We also compared microglial protein expression in AD with that in controls. Of interest, NEP level was higher in microglia in the AD group compared to the control group (Figure 2B).

**FIGURE 3 alz70339-fig-0003:**
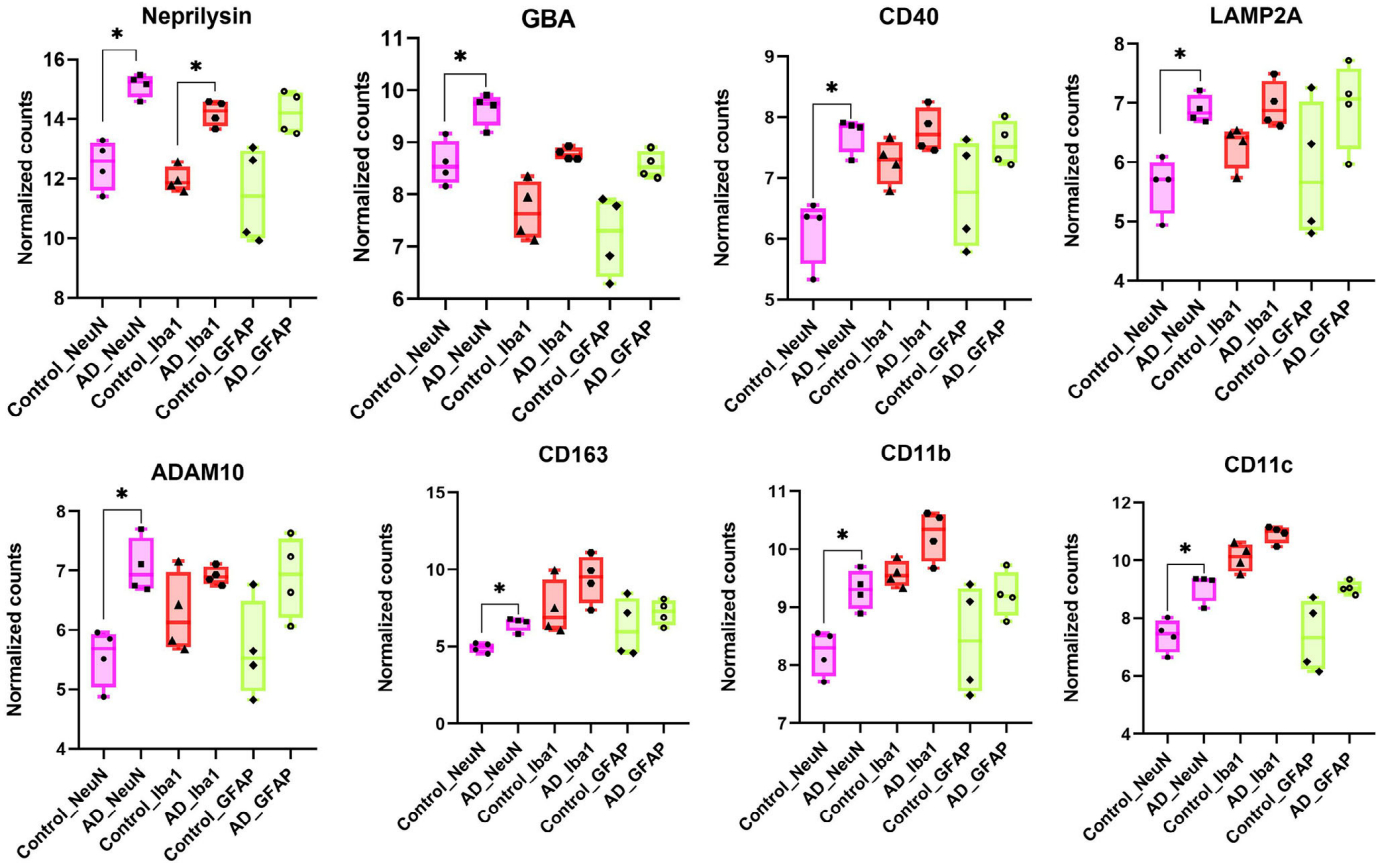
Box plots of significant protein targets in different cell types between AD and control (* *p*.adj ≤0.05). AD, Alzheimer's disease; GFAP, glial fibrillary acidic protein; NeuN, neuronal nuclei; Iba1, ionized calcium‐binding adapter molecule 1.

Figure 3 shows differential protein expression by cell type, which is also summarized in Tables S4–S6. AD brain phospho‐tau (S214) levels and phospho‐tau (S396) was higher in neurons (*p*.adj ≤  0.05). These findings are consistent with previous reports of phospho‐tau elevations in AD FFPE post‐mortem human brain proteomic studies.^29^, [Fig alz70339-fig-0002], [Fig alz70339-fig-0003]

NEP (an endopeptidase enzyme that plays a critical role as an Aβ peptide‐degrading enzyme^30^) was also higher with log fold changes of 2.66 and 2.23 in neurons and microglia, respectively (*p*.adj ≤ 0.05). Another significant protein alteration was GBA (a lysosomal enzyme that hydrolyzes the glycosidic linkage of glucocerebroside, a glycolipid in cell membranes^31^), with log fold changes of 1.05 in AD neurons compared to controls (*p*.adj ≤ 0.05).

We also identified LAMP2A as being higher in neurons in the AD cohort, might be indicative of chaperone‐mediated protein degradation being activated in neurons in AD. Neuroinflammatory markers CD11c, CD11b, and CD163 were also increased in neurons in AD compared to the control cohort. ADAM10 (a neuronal α‐secretase that proteolytically processes amyloid precursor protein [APP] along the non‐amyloidogenic pathway, generating neuroprotective fragments and mitigating Aβ plaque formation^32^) and CD40 (which plays an essential role in amyloid‐beta‐induced microglial activation^33^) were increased in AD brain neurons as well.

To further characterize the observed group differences, we quantified the variability of *APP* expression across the three studied cell types. APP was selected because it is a well‐established, consistently expressed protein in AD pathology,^34^, ^35^ making it a reliable internal reference. The mean ± SD values are presented in Table 3. Despite the small cohort size, the low SD observed in disease samples, particularly in neurons and microglia, suggests a consistent and narrow expression range for APP. This supports the expectation that variability will remain low even with larger sample sizes, strengthening confidence in the robustness of our pilot findings.

**TABLE 3:** APP expression variability by cell type in control and AD.

Cell type	Control (mean ± SD)	AD (mean ± SD)
Neurons	11.15 ± 0.66	11.96 ± 0.20
Microglia	11.68 ± 0.46	12.34 ± 0.24
Astrocytes	11.11 ± 1.18	11.97 ± 0.28

Abbreviations: AD, Alzheimer's disease; APP, amyloid precursor protein; SD, standard deviation.

To quantify the magnitude of group differences across the entire dataset, we calculated Cohen's *d* for each of the 76 proteins analyzed in neurons, microglia, and astrocytes.

For each protein, Cohen's *d* was calculated as:

$${\mathrm{Cohen}}^{\prime }s\;d=\left(M_{1}-M_{2}\right)/\surd \left(S{D_{1}}^{2}+S{D_{2}}^{2}\right)/2$$

where M₁ and M₂ represent the mean values of the control group and AD group, respectively, and SD₁ and SD₂ are the corresponding SDs.

**FIGURE 4:**
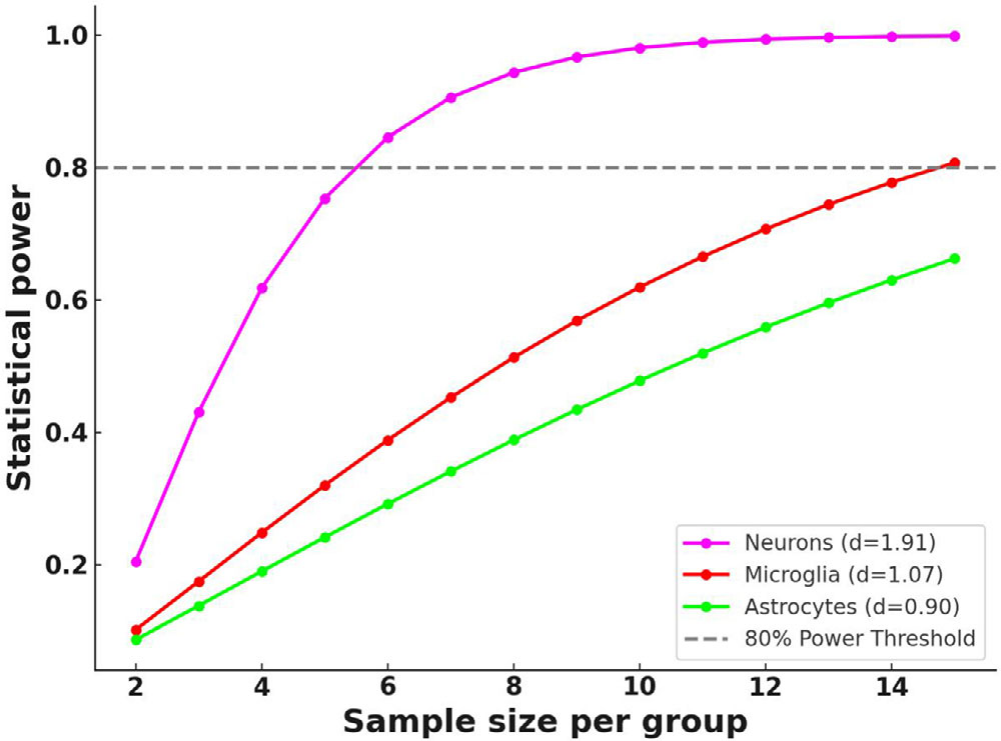
Power analysis across cell types and sample sizes.

The average effect sizes (Cohen's *d*) were 1.91 for neurons, 1.07 for microglia, and 0.90 for astrocytes. Based on these observed effect sizes and power calculations shown in Figure 4, we estimate that 5–6 samples per group are sufficient for neurons, whereas microglia and astrocytes require ≈10–15 samples per group to achieve 80% statistical power. These results help for the design of future studies building on these findings.

## 4 DISCUSSION

In this study, we compared protein expression between postmortem AD and non‐demented control brains utilizing the GeoMx DSP platform. We focused on protein expression profile of gray matter neurons, microglia, and astrocytes.

Quantitative analysis of Iba1‐positive cells demonstrated that 11% of the total cell population were microglia in the control group and 17% in the AD group. A review of the literature reveals adoption of Iba1 as a microglial marker presents conflicting result. Franco‐Bocanegra et al. showed a reduction in the number of ramified microglia and adoption of reactive morphology in the inferior parietal region without significant change in the number of total microglia.^36^ Similarly, the human post‐mortem study of Paasila et al. showed less ramified microglial cells in the AD inferior temporal cortex.^37^ Davies et al. observed no change in the density of microglial cell bodies in the inferior temporal and anterior cingulate cortices.^38^ These variations suggest that the response of different brain regions to microglial activation and loss might be heterogeneous, and a systematic study employing more than one microglial marker is needed.^37^, ^38^ One potential source of confounding that requires consideration is that non‐activate/quiescent microglia may elude detection using various platforms, including GeoMx, as a consequence of their small size and scant cytoplasm.

NEP, a metallopeptidase enzyme, plays a critical role in the breakdown of Aβ peptides. NEP has been shown to be particularly effective in cleaving Aβ42 (27%) and Aβ40 (73%) in their monomeric forms. However, mutations in *Aβ* can significantly hinder its susceptibility to NEP's proteolytic activity.^39^ Previous research suggests that increased NEP expression protects hippocampal neurons from Aβ‐induced toxicity in vitro. Increased NEP activity, therefore, could represent a potential strategy to protect the brain against the accumulation of Aβ and subsequent cognitive decline.^30^, ^40^, ^41^ Miners et al. reported a significant elevation in both unadjusted and neuron‐specific enolase (NSE)–adjusted NEP levels and activity within the AD group. Of interest, these NEP levels displayed a positive correlation with Braak stage (highly correlated with the disease severity) but a negative correlation with age in AD patients.^42^ Walker et al. also proposed a model where *PSEN1* and *BACE1* upregulation enhances APP processing, leading to increased Aβ production and deposition. Conversely, the upregulation of ADAM10, NEP, and insulin‐degrading enzyme (IDE) enzymes might be protective due to their role in Aβ degradation.^29^ However, it is important to note that Hellström‐Lindahl et al. did not observe any significant differences in NEP protein or mRNA levels in frontal and temporal cortex homogenates from AD and age‐matched control brains.^43^

Another protein whose levels were elevated in neurons in AD was GBA, a lysosomal enzyme essential for the degradation of glucosylceramide. Previous studies showed that mutants with GBA1 deficiency have been associated with neurodegenerative conditions including Parkinson's disease (PD) and dementia with Lewy bodies.^44^ However, little is known about GBA deficiency in AD. To our knowledge, only Choi et al. previously investigated GBA levels in postmortem human brain tissue. They demonstrated significantly decreased protein levels and enzyme activity in sporadic AD hippocampi, which suggests a potential compensatory mechanism or dysregulation. Choi et al. also observed that GBA facilitates the clearance of Aβ1‐42 oligomers and protects neurons from Aβ1‐42 oligomer‐induced cell death by enhancing lysosomal function.^45^

Our findings are also consistent with prior reports that identify lysosomal dysfunction in AD. In our study levels of LAMP2A were elevated in AD neurons. LAMP2A acts as a receptor in chaperone‐mediated autophagy (CMA), a cellular mechanism that specifically targets proteins for degradation by lysosomes. Reducing the expression of LAMP2A impairs the degradation of wild‐type tau protein, which suggests that lysosomes play a role in the degradation of wild‐type tau. In one study, blocking lysosomal proteolysis resulted in elevated tau levels, which argues that CMA, facilitated by LAMP2A function, plays a role in the lysosomal breakdown of wild‐type tau.^46^

We observed activation of neuroinflammation and chaperone protein degradation pathways in AD neurons, along with an upregulation of some protein targets in these cells. However, no significant change was seen in microglia and astrocytes. CD11b and CD11c were both found at higher levels in neurons, which is in line with previous reports.^47^

One particularly interesting study finding was that the ApoE protein was not significant in AD compared to non‐AD neurons. Brain ApoE protein production is typically associated with astrocytes and glia, although stressed neurons will also express ApoE.^48^

This study demonstrates the feasibility of using DSP technology and protein panels to spatially profile small numbers of FFPE post‐mortem brains. This specific technology does, however, have limitations. The presence of comorbidities in neurodegenerative diseases and the potential inaccuracies of neuropathological staging can reduce the fidelity of targeted reagents. The number of ROIs analyzed per slide is also restricted. Despite this, DSP technology still offers advantages over traditional methods, as it combines spatial and quantitative information that informs protein quantification within well‐defined tissue regions. Traditional immunohistochemistry offers qualitative protein identification but limited quantification, whereas methods such as Western blotting and proteomic mass spectrometry provide accurate quantification but lack spatial resolution. Finally, the small number of morphology markers may negatively impact assay sensitivity. Expanding the marker panel could help to better distinguish the brain's different cell types.

We acknowledge the limitations in estimating precise effect sizes from studies with small sample sizes. Nonetheless, the consistency observed in APP expression variability and the effect sizes calculated across multiple proteins suggest that the group differences reported here are robust. The relatively low variability, particularly within the disease groups, supports the reproducibility of these observations as sample sizes increase. The current dataset, although preliminary, offers valuable insights and serves as an informative pilot study. The power analysis performed based on observed effect sizes provides a rational framework for the design of future experiments, guiding sample size estimations to ensure adequate statistical power.

To conclude, in this pilot study, we investigated cell‐specific protein expression in the gray matter of AD brains compared to controls using DSP technology. Notably, we observed NEP upregulation across neurons and microglia. Although the study is limited by the small sample size typical of pilot‐scale analyses, the strength and consistency of the findings lay important groundwork for larger, more definitive studies. Future work will expand cohort sizes and incorporate complementary technologies, such as the CosMx Spatial Molecular Imager, to validate these molecular differences and further explore their mechanistic relevance to AD progression.

## AUTHOR CONTRIBUTIONS

Maryam Gholampour: data analysis, data interpretation, and writing the first draft of the manuscript. Malay K. Basu and Xinming Zhuo: data analysis and interpretation. Russell H. Swerdlow and Mohammad Haeri: study design, data interpretation, and study supervision. All authors contributed to critical revisions and approved the manuscript.

## CONFLICT OF INTEREST STATEMENT

The authors declare no conflicts of interest. Author disclosures are available in the Supporting Information.

## CONSENT STATEMENT

The Kansas University Medical Center Human Subjects Committee (KUMC HSC) issued approval for all involvement of human subjects, and all participants provided written informed consent prior to enrollment. This investigation was conducted in accordance with the Code of Ethics set forth by the World Medical Association (the Declaration of Helsinki).

## Supporting information



Supporting Information

Supporting Information

Supporting Information

## Data Availability

The complete dataset associated with this project has been deposited in Dryad under the https://doi.org/10.5061/dryad.w6m905r12. Lists of differentially expressed proteins are provided in Tables S4–S6.
