# Supplementary material for: Cell‐specific protein expression in Alzheimer's disease prefrontal cortex
Source: Alzheimers Dement. 2025 Jun 4;21(6):e70339. doi: 10.1002/alz.70339 (PMC12136086; doi:10.1002/alz.70339)
Supplement: Supplementary file 1 — Supporting Information [file ALZ-21-e70339-s003.docx]

**Table S1.** List of antibodies included in the core panel and module used in NanoString GeoMx DSP.

| **Protein Name** | **Gene** | **Accession** | **Full Target Name** | **Protein ID** |
| --- | --- | --- | --- | --- |
| Phospho-Tdp-43 (S409/S410) | TARDBP | Q13148, Q9H256, A0A024R4E2 | TAR DNA binding protein | DPROT_00115.1 |
| Tdp-43 | TARDBP | Q13148, Q9H256, A0A024R4E2 | TAR DNA binding protein | DPROT_00108.1 |
| Amyloid Precursor Protein | APP | B4DGD0, A0A0A0MRG2, B4DJT9, P05067, A0A140VJC8, E9PG40 | amyloid beta precursor protein | DPROT_00114.1 |
| Amyloid-Beta 1-40 | APP | B4DGD0, A0A0A0MRG2, B4DJT9, P05067, A0A140VJC8, E9PG40 | amyloid beta precursor protein | DPROT_00113.1 |
| Amyloid-Beta 1-42 | APP | B4DGD0, A0A0A0MRG2, B4DJT9, P05067, A0A140VJC8, E9PG40 | amyloid beta precursor protein | DPROT_00116.1 |
| APOE | APOE | A0A0S2Z3D5, P02649 | apolipoprotein E | DPROT_00109.1 |
| P2RX7 | P2RX7 | Q99572 | purinergic receptor P2X 7 | DPROT_00142.1 |
| Phospho-Tau (S404) | MAPT | A0A024R9Y0, A0A024RA17, B3KTM0, A0A024R9Y1, P10636, A0A024RA19 | microtubule associated protein tau | DPROT_00111.1 |
| Tau | MAPT | A0A024R9Y0, A0A024RA17, B3KTM0, A0A024R9Y1, P10636, A0A024RA19 | microtubule associated protein tau | DPROT_00110.1 |
| Ubiquitin | UBB | P0CG47, Q5U5U6 | ubiquitin B | DPROT_00117.1 |
| PSEN1 | PSEN1 | A0A024R6A3, A0A0S2Z4D2, P49768 | presenilin 1 | DPROT_00173.1 |
| ADAM10 | ADAM10 | A0A024R5U5, O14672 | ADAM metallopeptidase domain 10 | DPROT_00140.1 |
| BACE1 | BACE1 | A0A024R3D7, P56817, B7Z3K2, A0A024R3E8, A0A024R3F9, B7Z3Z4, Q5W9H2, A0A024R3D5 | beta-secretase 1 | DPROT_00172.1 |
| IDE | IDE | P14735, B3KSB8 | insulin degrading enzyme | DPROT_00145.1 |
| Neprilysin | MME | P08473 | membrane metalloendopeptidase | DPROT_00144.1 |
| NRGN | NRGN | Q92686, A0A024R3M7 | neurogranin | DPROT_00143.1 |
| Phospho-Tau (S199) | MAPT | A0A024R9Y0, A0A024RA17, B3KTM0, A0A024R9Y1, P10636, A0A024RA19 | microtubule associated protein tau | DPROT_00139.1 |
| Phospho-Tau (S214) | MAPT | A0A024R9Y0, A0A024RA17, B3KTM0, A0A024R9Y1, P10636, A0A024RA19 | microtubule associated protein tau | DPROT_00141.1 |
| Phospho-Tau (S396) | MAPT | A0A024R9Y0, A0A024RA17, B3KTM0, A0A024R9Y1, P10636, A0A024RA19 | microtubule associated protein tau | DPROT_00138.1 |
| Phospho-Tau (T231) | MAPT | A0A024R9Y0, A0A024RA17, B3KTM0, A0A024R9Y1, P10636, A0A024RA19 | microtubule associated protein tau | DPROT_00137.1 |
| ATG5 | ATG5 | A9UGY9, Q7Z3H3, Q9H1Y0 | autophagy related 5 | DPROT_00277.1 |
| LAMP2A | LAMP2 | P13473 | lysosomal associated membrane protein 2 | DPROT_00282.1 |
| ATG12 | ATG12 | O94817 | autophagy related 12 | DPROT_00276.1 |
| BAG3 | BAG3 | O95817 | BAG cochaperone 3 | DPROT_00278.1 |
| GBA | GBA | B7Z6S9, P04062, A0A068F658 | glucosylceramidase beta | DPROT_00280.1 |
| HSC70 | HSPA8 | P11142, V9HW22, Q53HF2 | heat shock protein family A (Hsp70) member 8 | DPROT_00281.1 |
| LC3B | MAP1LC3B | Q9GZQ8, Q658J6 | microtubule associated protein 1 light chain 3 beta | DPROT_00295.1 |
| P62 | SQSTM1 | Q13501 | sequestosome 1 | DPROT_00298.1 |
| TFEB | TFEB | P19484, B0QYS6, A0A024RCY3 | transcription factor EB | DPROT_00283.1 |
| VPS35 | VPS35 | Q96QK1 | VPS35 retromer complex component | DPROT_00285.1 |
| CLEC7A | CLEC7A | Q9BXN2, Q68D78, A0A024RAN9, A0A0S2Z5Q1 | C-type lectin domain containing 7A | DPROT_00341.1 |
| C4B | C4B | P0C0L4, P0C0L5 | complement C4B (Chido blood group) | DPROT_00287.1 |
| CD11c | ITGAX | H3BN02, P20702 | integrin subunit alpha X | DPROT_00011.1 |
| CD9 | CD9 | A6NNI4, B4DK09, P21926 | CD9 molecule | DPROT_00289.1 |
| CSF1R | CSF1R | P07333 | colony stimulating factor 1 receptor | DPROT_00291.1 |
| CTSD | CTSD | P07339, V9HWI3 | cathepsin D | DPROT_00292.1 |
| EMP1 | EMP1 | P54849, A0A024RAT0 | epithelial membrane protein 1 | DPROT_00344.1 |
| GPNMB | GPNMB | Q14956, Q96F58, A0A024RA55 | glycoprotein nmb | DPROT_00206.1 |
| MERTK | MERTK | Q12866 | MER proto-oncogene, tyrosine kinase | DPROT_00293.1 |
| Vimentin | VIM | V9HWE1, P08670 | vimentin | DPROT_00168.1 |
| CD40 | CD40 | A0A0S2Z3C7, Q6P2H9, P25942, A0A0S2Z349 | CD40 molecule | DPROT_00042.1 |
| Olig2 | OLIG2 | Q13516 | oligodendrocyte transcription factor 2 | DPROT_00102.1 |
| Ms IgG1 |  |  |  | DPROT_00002.1 |
| Ms IgG2a |  |  |  | DPROT_00003.1 |
| Rb IgG |  |  |  | DPROT_00001.1 |
| GAPDH | GAPDH | P04406, V9HVZ4 | glyceraldehyde-3-phosphate dehydrogenase | DPROT_00020.1 |
| Histone H3 | H3C1 | P68431 | H3 clustered histone 1 | DPROT_00005.1 |
| S6 | RPS6 | P62753, A2A3R6 | ribosomal protein S6 | DPROT_00008.1 |
| CD11b | ITGAM | P11215 | integrin subunit alpha M | DPROT_00095.1 |
| CD163 | CD163 | Q86VB7 | CD163 molecule | DPROT_00052.1 |
| CD31 | PECAM1 | P16284, A0A075B738 | platelet and endothelial cell adhesion molecule 1 | DPROT_00104.1 |
| CD39 | ENTPD1 | P49961 | ectonucleoside triphosphate diphosphohydrolase 1 | DPROT_00105.1 |
| CD45 | PTPRC | P08575, X6R433, M9MML4, A0A0A0MT22 | protein tyrosine phosphatase receptor type C | DPROT_00015.1 |
| CD68 | CD68 | P34810 | CD68 molecule | DPROT_00006.1 |
| GFAP | GFAP | P14136, K7EMP8, A7REI1 | glial fibrillary acidic protein | DPROT_00099.1 |
| HLA-DR | HLA-DRA | A0A0G2JMH6, P01903 | major histocompatibility complex, class II, DR alpha | DPROT_00007.1 |
| IBA1 | AIF1 | Q4V347, P55008, I3WTX1 | allograft inflammatory factor 1 | DPROT_00098.1 |
| Ki-67 | MKI67 | P46013 | marker of proliferation Ki-67 | DPROT_00009.1 |
| MAP2 | MAP2 | A0A024R409, A0A024R407, P11137, A0A024R3Z1, Q6NYC5, A0A024R3Y8 | microtubule associated protein 2 | DPROT_00096.1 |
| Myelin basic protein | MBP | A0A024R384, P02686 | myelin basic protein | DPROT_00100.1 |
| NeuN | RBFOX3 | A6NFN3 | RNA binding fox-1 homolog 3 | DPROT_00101.1 |
| Neurofilament light | NEFL | P07196 | neurofilament light chain | DPROT_00103.1 |
| P2ry12 | P2RY12 | A8K7T1, Q9H244 | purinergic receptor P2Y12 | DPROT_00106.1 |
| S100B | S100B | A0A0S2Z4C5, P04271 | S100 calcium binding protein B | DPROT_00056.1 |
| Synaptophysin | SYP | P08247 | synaptophysin | DPROT_00112.1 |
| TMEM119 | TMEM119 | Q4V9L6 | transmembrane protein 119 | DPROT_00107.1 |
| FUS | FUS | Q6IBQ5, P35637, Q13344 | FUS RNA binding protein | DPROT_00127.1 |
| Phospho-Alpha-synuclein (S129) | SNCA | H6UYS5, P37840 | synuclein alpha | DPROT_00119.1 |
| PINK1 | PINK1 | Q9BXM7 | PTEN induced kinase 1 | DPROT_00126.1 |
| Alpha-synuclein | SNCA | H6UYS5, P37840 | synuclein alpha | DPROT_00535.1 |
| ApoA-I | APOA1 | A0A024R3E3, P02647 | apolipoprotein A1 | DPROT_00120.1 |
| Calbindin | CALB1 | P05937 | calbindin 1 | DPROT_00125.1 |
| LRRK2 | LRRK2 | Q17RV3, Q5S007 | leucine rich repeat kinase 2 | DPROT_00121.1 |
| Park5 | UCHL1 | V9HW74, P09936 | ubiquitin C-terminal hydrolase L1 | DPROT_00122.1 |
| Park7 | PARK7 | V9HWC2, Q99497 | Parkinsonism associated deglycase | DPROT_00123.1 |
| Tyrosine Hydroxylase | TH | P78428, P07101 | tyrosine hydroxylase | DPROT_00124.1 |

None of the proteins in this study showed a significant decrease in expression levels. The issue arises from the limited number of cells in the selected ROI. This limitation can be attributed to the insufficient number of cells within the selected ROI required by NanoString GeoMx DSP. While increases in protein expression are readily detectable, decreases may fall below the sensitivity threshold required for accurate identification. The higher sensitivity for detecting upregulation relative to downregulation is explained by this difference in detection power. Furthermore, in AD, the number of cells or protein level may be diminished, particularly in neurons. This reduction in cell count/protein level can result in lower detection within the same area. Consequently, if a protein is initially expressed at a low level, it may eventually fall below our detection sensitivity. A similar issue with reduced sensitivity in detecting decreased protein levels using NanoString GeoMx DSP has been observed in other studies on AD [1] and other conditions [2, 3]. To address this limitation, a substantially larger ROI is necessary to enhance the ability to detect downregulated proteins.

**Table S2.** Details of antibodies used in multiplex immunofluorescence staining.

|  |  | **Antibody** | **Host** | **Dilution factor used** | **Manufactured by** | **Clone** |
| --- | --- | --- | --- | --- | --- | --- |
| 1 |  | NeuN | Rabbit | 100 | Millipore Sigma | Polyclonal |
| 2 |  | GFAP | Mouse | 400 | Novus Bio | GA5 |
| 3 |  | Iba1 | Rabbit | 100 | Cell Signaling Technology | E404W |

**Table S3.** Classification of Protein Targets from Nanostring Human Neuroscience Panel.

| **Function** | **Investigated Targets** |
| --- | --- |
| Chaperone/Protein Degradation | Ubiquitin [4], HSC70 [5], BAG3 [6], P62 [7], TFEB [8], LAMP2A [9], GPNMB [10], VPS35 [11], ATG5, ATG12 [12] |
| Immune Response | CD31 [13], CD39 [14], CD45 [15], C4B [16] |
| Neuroinflammation | P2RX7 [17], CD163 [18], CD68 [19], GFAP [20], CSF1R [21], CD11b, CD11c [22] |
| Protease | Neprilysin [23], IDE [24], BACE1 [25], ADAM10 [26], CTSD [27], APOE [28], PSEN1 [29], Amyloid Precursor Protein, Amyloid-Beta 1-40, Amyloid-Beta 1-42 [30] |
| RNA Processing | TDP-43, Phospho-Tdp-43 (S409/S410) [31], FUS [32] |
| Metabolism and mitochondrial function | APOE [33], ApoA-I [34], PINK1 [35] |
| Autophagy Promotion | ATG5, ATG12 [36], LAMP2A, HSC70 [37], BAG3 [6], GBA [37], LC3B [38], P62 [39] , TFEB [40], VPS35 [41] |
| Cytoskeleton | Neurofilament light [42], MAP2 [43], EMP1 [44], Tau, Phospho-Tau (S404), Phospho-Tau (S199), Phospho-Tau (S214), Phospho-Tau (S396), Phospho-Tau (T231) [45] |
| Microglial activation | CLEC7A, TMEM119 [46], CD40, CD45 [47], Vimentin [48], Iba1 [49], P2RX7 [50], CD68 [19], CD11b, CD11c [22], CD163 [51], MERTK [52] |
| Parkinson's Disease-Related Proteins | Alpha-synuclein [53], Phospho-Alpha-synuclein (S129) [54], LRRK2 [55], Park5, Park7 [56] |
| Neuronal Markers | NeuN, MAP2, Synaptophysin [57], NRGN [58], Calbindin [59] |
| Oligodendrocyte Markers | Myelin basic protein, olig2 [60] |
| Additional proteins | Ki-67 [61], Tyrosine Hydroxylase [62] |
| Reference proteins | Ms IgG1, Ms IgG2a, Rb IgG, GAPDH, Histone H3, S6 |

References:

[1] Walker JM, Kazempour Dehkordi S, Fracassi A, Vanschoiack A, Pavenko A, Taglialatela G, et al. Differential protein expression in the hippocampi of resilient individuals identified by digital spatial profiling. Acta Neuropathol Commun. 2022;10:23.

[2] Yang DC, Hsu SW, Li JM, Oldham J, Chen CH. Spatial Decoding of Immune Cell Contribution to Fibroblastic Foci in Idiopathic Pulmonary Fibrosis. Am J Respir Crit Care Med. 2023;208:728-31.

[3] Nevarez-Mejia J, Pickering H, Sosa RA, Valenzuela NM, Fishbein GA, Baldwin WM, 3rd, et al. Spatial multiomics of arterial regions from cardiac allograft vasculopathy rejected grafts reveal novel insights into the pathogenesis of chronic antibody-mediated rejection. Am J Transplant. 2024;24:1146-60.

[4] Gadhave K, Bolshette N, Ahire A, Pardeshi R, Thakur K, Trandafir C, et al. The ubiquitin proteasomal system: a potential target for the management of Alzheimer's disease. J Cell Mol Med. 2016;20:1392-407.

[5] Koren J, 3rd, Jinwal UK, Lee DC, Jones JR, Shults CL, Johnson AG, et al. Chaperone signalling complexes in Alzheimer's disease. J Cell Mol Med. 2009;13:619-30.

[6] Morawe T, Hiebel C, Kern A, Behl C. Protein homeostasis, aging and Alzheimer's disease. Mol Neurobiol. 2012;46:41-54.

[7] Liu WJ, Ye L, Huang WF, Guo LJ, Xu ZG, Wu HL, et al. p62 links the autophagy pathway and the ubiqutin-proteasome system upon ubiquitinated protein degradation. Cell Mol Biol Lett. 2016;21:29.

[8] Martini-Stoica H, Xu Y, Ballabio A, Zheng H. The Autophagy-Lysosomal Pathway in Neurodegeneration: A TFEB Perspective. Trends Neurosci. 2016;39:221-34.

[9] Rout AK, Strub MP, Piszczek G, Tjandra N. Structure of transmembrane domain of lysosome-associated membrane protein type 2a (LAMP-2A) reveals key features for substrate specificity in chaperone-mediated autophagy. J Biol Chem. 2014;289:35111-23.

[10] Budge KM, Neal ML, Richardson JR, Safadi FF. Glycoprotein NMB: an Emerging Role in Neurodegenerative Disease. Mol Neurobiol. 2018;55:5167-76.

[11] Filippone A, Pratico D, Esposito E. VPS35 down regulation alters degradation pathways in neuronal cells. Alzheimer's & Dementia. 2021;17:e057798.

[12] Haller M, Hock AK, Giampazolias E, Oberst A, Green DR, Debnath J, et al. Ubiquitination and proteasomal degradation of ATG12 regulates its proapoptotic activity. Autophagy. 2014;10:2269-78.

[13] Zhang Z, Gan Q, Han J, Tao Q, Qiu WQ, Madri JA. CD31 as a probable responding and gate-keeping protein of the blood-brain barrier and the risk of Alzheimer's disease. J Cereb Blood Flow Metab. 2023;43:1027-41.

[14] Antonioli L, Pacher P, Vizi ES, Haskó G. CD39 and CD73 in immunity and inflammation. Trends Mol Med. 2013;19:355-67.

[15] Zhu Y, Hou H, Rezai-Zadeh K, Giunta B, Ruscin A, Gemma C, et al. CD45 deficiency drives amyloid-β peptide oligomers and neuronal loss in Alzheimer's disease mice. J Neurosci. 2011;31:1355-65.

[16] Zorzetto M, Datturi F, Divizia L, Pistono C, Campo I, De Silvestri A, et al. Complement C4A and C4B Gene Copy Number Study in Alzheimer's Disease Patients. Curr Alzheimer Res. 2017;14:303-8.

[17] Chen Y-H, Lin R-R, Tao Q-Q. The role of P2X7R in neuroinflammation and implications in Alzheimer's disease. Life Sciences. 2021;271:119187.

[18] Etzerodt A, Moestrup SK. CD163 and inflammation: biological, diagnostic, and therapeutic aspects. Antioxid Redox Signal. 2013;18:2352-63.

[19] Leng F, Edison P. Neuroinflammation and microglial activation in Alzheimer disease: where do we go from here? Nature Reviews Neurology. 2021;17:157-72.

[20] Azzolini F, Gilio L, Pavone L, Iezzi E, Dolcetti E, Bruno A, et al. Neuroinflammation Is Associated with GFAP and sTREM2 Levels in Multiple Sclerosis. Biomolecules. 2022;12.

[21] Chitu V, Stanley ER. Colony-stimulating factor-1 in immunity and inflammation. Current Opinion in Immunology. 2006;18:39-48.

[22] Kamphuis W, Kooijman L, Schetters S, Orre M, Hol EM. Transcriptional profiling of CD11c-positive microglia accumulating around amyloid plaques in a mouse model for Alzheimer's disease. Biochimica et Biophysica Acta (BBA) - Molecular Basis of Disease. 2016;1862:1847-60.

[23] Grimm MO, Mett J, Stahlmann CP, Haupenthal VJ, Zimmer VC, Hartmann T. Neprilysin and Aβ Clearance: Impact of the APP Intracellular Domain in NEP Regulation and Implications in Alzheimer's Disease. Front Aging Neurosci. 2013;5:98.

[24] Farris W, Mansourian S, Chang Y, Lindsley L, Eckman EA, Frosch MP, et al. Insulin-degrading enzyme regulates the levels of insulin, amyloid beta-protein, and the beta-amyloid precursor protein intracellular domain in vivo. Proc Natl Acad Sci U S A. 2003;100:4162-7.

[25] Hampel H, Vassar R, De Strooper B, Hardy J, Willem M, Singh N, et al. The β-Secretase BACE1 in Alzheimer's Disease. Biol Psychiatry. 2021;89:745-56.

[26] Yuan XZ, Sun S, Tan CC, Yu JT, Tan L. The Role of ADAM10 in Alzheimer's Disease. J Alzheimers Dis. 2017;58:303-22.

[27] Gallwitz L, Schmidt L, Marques ARA, Tholey A, Cassidy L, Ulku I, et al. Cathepsin D: Analysis of its potential role as an amyloid beta degrading protease. Neurobiol Dis. 2022;175:105919.

[28] Jiang Q, Lee CY, Mandrekar S, Wilkinson B, Cramer P, Zelcer N, et al. ApoE promotes the proteolytic degradation of Abeta. Neuron. 2008;58:681-93.

[29] De Strooper B, Iwatsubo T, Wolfe MS. Presenilins and γ-secretase: structure, function, and role in Alzheimer Disease. Cold Spring Harb Perspect Med. 2012;2:a006304.

[30] O'Brien RJ, Wong PC. Amyloid precursor protein processing and Alzheimer's disease. Annu Rev Neurosci. 2011;34:185-204.

[31] Meneses A, Koga S, O’Leary J, Dickson DW, Bu G, Zhao N. TDP-43 Pathology in Alzheimer’s Disease. Molecular Neurodegeneration. 2021;16:84.

[32] Deng H, Gao K, Jankovic J. The role of FUS gene variants in neurodegenerative diseases. Nature Reviews Neurology. 2014;10:337-48.

[33] Gabrielli AP, Weidling I, Ranjan A, Wang X, Novikova L, Chowdhury SR, et al. Mitochondria Profoundly Influence Apolipoprotein E Biology. J Alzheimers Dis. 2023;92:591-604.

[34] Pedrini S, Chatterjee P, Hone E, Martins RN. High-density lipoprotein-related cholesterol metabolism in Alzheimer’s disease. Journal of Neurochemistry. 2021;159:343-77.

[35] Zhou TY, Ma RX, Li J, Zou B, Yang H, Ma RY, et al. Review of PINK1-Parkin-mediated mitochondrial autophagy in Alzheimer's disease. Eur J Pharmacol. 2023;959:176057.

[36] Longobardi A, Catania M, Geviti A, Salvi E, Vecchi ER, Bellini S, et al. Autophagy Markers Are Altered in Alzheimer’s Disease, Dementia with Lewy Bodies and Frontotemporal Dementia. International Journal of Molecular Sciences2024.

[37] Wang YT, Lu JH. Chaperone-Mediated Autophagy in Neurodegenerative Diseases: Molecular Mechanisms and Pharmacological Opportunities. Cells. 2022;11.

[38] Martinez J, Almendinger J, Oberst A, Ness R, Dillon CP, Fitzgerald P, et al. Microtubule-associated protein 1 light chain 3 alpha (LC3)-associated phagocytosis is required for the efficient clearance of dead cells. Proceedings of the National Academy of Sciences. 2011;108:17396-401.

[39] Ma S, Attarwala IY, Xie X-Q. SQSTM1/p62: A Potential Target for Neurodegenerative Disease. ACS Chemical Neuroscience. 2019;10:2094-114.

[40] Song TT, Cai RS, Hu R, Xu YS, Qi BN, Xiong YA. The important role of TFEB in autophagy-lysosomal pathway and autophagy-related diseases: a systematic review. Eur Rev Med Pharmacol Sci. 2021;25:1641-9.

[41] Zavodszky E, Seaman MN, Moreau K, Jimenez-Sanchez M, Breusegem SY, Harbour ME, et al. Mutation in VPS35 associated with Parkinson's disease impairs WASH complex association and inhibits autophagy. Nat Commun. 2014;5:3828.

[42] Zhao Y, Arceneaux L, Culicchia F, Lukiw WJ. Neurofilament Light (NF-L) Chain Protein from a Highly Polymerized Structural Component of the Neuronal Cytoskeleton to a Neurodegenerative Disease Biomarker in the Periphery. HSOA J Alzheimers Neurodegener Dis. 2021;7.

[43] DeGiosio RA, Grubisha MJ, MacDonald ML, McKinney BC, Camacho CJ, Sweet RA. More than a marker: potential pathogenic functions of MAP2. Front Mol Neurosci. 2022;15:974890.

[44] Walker DG, Link J, Lue L-F, Dalsing-Hernandez JE, Boyes BE. Gene expression changes by amyloid β peptide-stimulated human postmortem brain microglia identify activation of multiple inflammatory processes. Journal of Leukocyte Biology. 2006;79:596-610.

[45] Medeiros R, Baglietto-Vargas D, LaFerla FM. The role of tau in Alzheimer's disease and related disorders. CNS Neurosci Ther. 2011;17:514-24.

[46] Hansen DV, Hanson JE, Sheng M. Microglia in Alzheimer's disease. J Cell Biol. 2018;217:459-72.

[47] Walker D, Lue L-F. Immune phenotypes of microglia in human neurodegenerative disease: Challenges to detecting microglial polarization in human brains. Alzheimer's research & therapy. 2015;7:56.

[48] Yamada T, Kawamata T, Walker DG, McGeer PL. Vimentin immunoreactivity in normal and pathological human brain tissue. Acta Neuropathol. 1992;84:157-62.

[49] Tischer J, Krueger M, Mueller W, Staszewski O, Prinz M, Streit WJ, et al. Inhomogeneous distribution of Iba-1 characterizes microglial pathology in Alzheimer's disease. Glia. 2016;64:1562-72.

[50] Territo PR, Zarrinmayeh H. P2X(7) Receptors in Neurodegeneration: Potential Therapeutic Applications From Basic to Clinical Approaches. Front Cell Neurosci. 2021;15:617036.

[51] Pey P, Pearce RK, Kalaitzakis ME, Griffin WS, Gentleman SM. Phenotypic profile of alternative activation marker CD163 is different in Alzheimer's and Parkinson's disease. Acta Neuropathol Commun. 2014;2:21.

[52] Shen K, Reichelt M, Kyauk RV, Ngu H, Shen YA, Foreman O, et al. Multiple sclerosis risk gene Mertk is required for microglial activation and subsequent remyelination. Cell Rep. 2021;34:108835.

[53] Recasens A, Dehay B. Alpha-synuclein spreading in Parkinson's disease. Front Neuroanat. 2014;8:159.

[54] Oueslati A. Implication of Alpha-Synuclein Phosphorylation at S129 in Synucleinopathies: What Have We Learned in the Last Decade? J Parkinsons Dis. 2016;6:39-51.

[55] Rui Q, Ni H, Li D, Gao R, Chen G. The Role of LRRK2 in Neurodegeneration of Parkinson Disease. Curr Neuropharmacol. 2018;16:1348-57.

[56] Dawson TM, Dawson VL. The role of parkin in familial and sporadic Parkinson's disease. Mov Disord. 2010;25 Suppl 1:S32-9.

[57] Gusel'nikova VV, Korzhevskiy DE. NeuN As a Neuronal Nuclear Antigen and Neuron Differentiation Marker. Acta Naturae. 2015;7:42-7.

[58] Xiang Y, Xin J, Le W, Yang Y. Neurogranin: A Potential Biomarker of Neurological and Mental Diseases. Front Aging Neurosci. 2020;12:584743.

[59] Hof PR, Morrison JH. Neocortical neuronal subpopulations labeled by a monoclonal antibody to calbindin exhibit differential vulnerability in Alzheimer's disease. Experimental Neurology. 1991;111:293-301.

[60] Huang H, He W, Tang T, Qiu M. Immunological Markers for Central Nervous System Glia. Neurosci Bull. 2023;39:379-92.

[61] Smith TW, Lippa CF. Ki-67 immunoreactivity in Alzheimer's disease and other neurodegenerative disorders. J Neuropathol Exp Neurol. 1995;54:297-303.

[62] Nagatsu T, Nakashima A, Ichinose H, Kobayashi K. Human tyrosine hydroxylase in Parkinson's disease and in related disorders. J Neural Transm (Vienna). 2019;126:397-409.
