# Supplementary material for: Cell‐specific protein expression in Alzheimer's disease prefrontal cortex
Source: Alzheimers Dement. 2025 Jun 4;21(6):e70339. doi: 10.1002/alz.70339 (PMC12136086; doi:10.1002/alz.70339)
Supplement: Supplementary file 2 — Supporting Information [file ALZ-21-e70339-s002.docx]

**Table S4. p-value sheet of neurons (NeuN)**

|  | | | | | | | | | | | | | |  |
| --- | --- | --- | --- | --- | --- | --- | --- | --- | --- | --- | --- | --- | --- | --- |
|  | **Ctl_NeuN_1** | **Ctl_NeuN_2** | **Ctl_NeuN_3** | **Ctl_NeuN_4** | **AD_NeuN_5** | **AD_NeuN_6** | **AD_NeuN_7** | **AD_NeuN_8** | **log Fold Change** | **mean_Ctl** | **mean_AD** | **t_test_p_val** | **p.adj** |  |
| **Phospho-Tau (S214)** | 6.546819 | 4.944783 | 5.49498 | 5.781284 | 12.18083 | 11.36445 | 9.629925 | 10.09459 | 5.125482 | 5.691966 | 10.81745 | 0.000760234 | 0.036568358 |  |
| **CD163** | 4.528429 | 4.808537 | 5.147339 | 5.212927 | 6.789424 | 6.637421 | 6.694565 | 5.815419 | 1.559899 | 4.924308 | 6.484207 | 0.00186492 | 0.036568358 |  |
| **Rb IgG** | 6.144397 | 6.926396 | 6.798072 | 6.831239 | 7.78722 | 7.626397 | 7.991234 | 8.183879 | 1.222156 | 6.675026 | 7.897183 | 0.002019952 | 0.036568358 |  |
| **LC3B** | 10.12572 | 10.45121 | 10.20734 | 9.751658 | 11.27772 | 11.10157 | 11.05956 | 10.85583 | 0.939686 | 10.13398 | 11.07367 | 0.002751211 | 0.036568358 |  |
| **Phospho-Tdp-43 (S409/S410)** | 4.951269 | 5.56794 | 5.598967 | 4.998574 | 6.720572 | 6.154974 | 6.283708 | 6.229936 | 1.06811 | 5.279188 | 6.347297 | 0.00346014 | 0.036568358 |  |
| **Neprilysin** | 11.40349 | 12.24045 | 12.93488 | 13.28297 | 15.47413 | 15.31445 | 15.16745 | 14.58519 | 2.669856 | 12.46545 | 15.1353 | 0.003572207 | 0.036568358 |  |
| **ADAM10** | 5.85225 | 5.957219 | 5.516647 | 4.879217 | 7.700661 | 7.112559 | 6.749989 | 6.688061 | 1.511484 | 5.551333 | 7.062817 | 0.004139018 | 0.036568358 |  |
| **CD11c** | 6.649343 | 7.357163 | 8.027855 | 7.583755 | 9.364785 | 8.350979 | 9.37027 | 9.322964 | 1.69772 | 7.404529 | 9.102249 | 0.004531496 | 0.036568358 |  |
| **CD40** | 5.333386 | 6.551291 | 6.364413 | 6.348982 | 7.913675 | 7.290608 | 7.836432 | 7.865001 | 1.576911 | 6.149518 | 7.726429 | 0.005074532 | 0.036568358 |  |
| **CD39** | 4.921257 | 4.50622 | 4.705528 | 4.336295 | 6.01877 | 5.488255 | 6.01877 | 5.225221 | 1.070429 | 4.617325 | 5.687754 | 0.005862911 | 0.036568358 |  |
| **CD11b** | 7.711981 | 8.553454 | 8.503557 | 8.095554 | 9.697663 | 8.892311 | 9.219831 | 9.398103 | 1.085841 | 8.216137 | 9.301977 | 0.006050485 | 0.036568358 |  |
| **CD31** | 7.628852 | 8.43833 | 8.252941 | 7.977307 | 9.307529 | 8.835948 | 8.990884 | 8.848475 | 0.921351 | 8.074358 | 8.995709 | 0.006689777 | 0.036568358 |  |
| **Phospho-Tau (S396)** | 4.894418 | 7.981881 | 8.971862 | 8.403145 | 13.59134 | 12.29345 | 12.21172 | 12.9203 | 5.191377 | 7.562827 | 12.7542 | 0.007070253 | 0.036568358 |  |
| **Myelin basic protein** | 14.69039 | 13.48188 | 14.08493 | 13.70798 | 15.43464 | 15.67403 | 14.78281 | 15.55078 | 1.36927 | 13.99129 | 15.36057 | 0.007113682 | 0.036568358 |  |
| **LAMP2A** | 4.938037 | 6.092365 | 5.711761 | 5.711761 | 7.216105 | 6.906149 | 6.687726 | 6.75547 | 1.277882 | 5.613481 | 6.891363 | 0.007372784 | 0.036568358 |  |
| **CD68** | 7.381266 | 8.082232 | 8.207062 | 8.098959 | 9.024256 | 8.52925 | 8.916933 | 9.106718 | 0.951909 | 7.94238 | 8.894289 | 0.007797345 | 0.036568358 |  |
| **Alpha-synuclein** | 12.99393 | 14.48913 | 13.88689 | 13.5249 | 16.17357 | 15.14688 | 14.99468 | 15.10555 | 1.631457 | 13.72371 | 15.35517 | 0.008179764 | 0.036568358 |  |
| **GBA** | 8.636516 | 9.163861 | 8.422972 | 8.158814 | 9.904796 | 9.713156 | 9.777985 | 9.1911 | 1.051218 | 8.595541 | 9.646759 | 0.008725683 | 0.036841773 |  |
| **HLA-DR** | 6.938181 | 7.968427 | 7.706589 | 7.284356 | 8.607041 | 8.23484 | 8.487606 | 8.630564 | 1.015624 | 7.474388 | 8.490013 | 0.014856087 | 0.055597109 |  |
| **TMEM119** | 8.083486 | 9.373472 | 9.654339 | 9.31861 | 10.90943 | 10.27638 | 10.65552 | 10.79144 | 1.550717 | 9.107477 | 10.65819 | 0.015172153 | 0.055597109 |  |
| **Ubiquitin** | 9.026637 | 9.381232 | 9.585672 | 8.860493 | 11.50832 | 10.14754 | 10.18564 | 10.46509 | 1.36314 | 9.213509 | 10.57665 | 0.015362359 | 0.055597109 |  |
| **CD45** | 7.795102 | 8.868759 | 8.658285 | 8.835744 | 9.903846 | 9.262501 | 9.627683 | 9.592769 | 1.057227 | 8.539473 | 9.5967 | 0.016558576 | 0.057202353 |  |
| **Amyloid-Beta 1-42** | 8.828112 | 9.759339 | 9.391354 | 8.924328 | 11.00169 | 10.05202 | 9.984073 | 10.09399 | 1.057159 | 9.225783 | 10.28294 | 0.017365767 | 0.057382535 |  |
| **APOE** | 11.08165 | 10.59281 | 11.31077 | 9.476433 | 12.77096 | 12.33331 | 12.03429 | 11.95398 | 1.657718 | 10.61542 | 12.27313 | 0.019261008 | 0.060993192 |  |
| **Olig2** | 6.910306 | 8.088903 | 8.498992 | 7.882072 | 8.962966 | 8.95147 | 9.474865 | 9.397903 | 1.351733 | 7.845068 | 9.196801 | 0.020733385 | 0.063029491 |  |
| **Ms IgG1** | 6.357971 | 7.044813 | 6.714665 | 6.570275 | 7.307752 | 7.007519 | 7.307752 | 7.179835 | 0.528784 | 6.671931 | 7.200714 | 0.026512401 | 0.077497787 |  |
| **GAPDH** | 13.95915 | 14.91974 | 14.56464 | 14.02547 | 17.22373 | 15.60507 | 15.48367 | 15.38398 | 1.556862 | 14.36725 | 15.92411 | 0.028557878 | 0.080385137 |  |
| **GPNMB** | 4.801767 | 5.837391 | 6.094549 | 6.003401 | 8.149697 | 5.98531 | 7.702238 | 7.559175 | 1.664828 | 5.684277 | 7.349105 | 0.030190185 | 0.081944788 |  |
| **Phospho-Tau (T231)** | 6.37677 | 7.873196 | 7.897342 | 7.435664 | 8.996525 | 8.479467 | 8.307986 | 8.358451 | 1.139864 | 7.395743 | 8.535607 | 0.04116576 | 0.107426164 |  |
| **Park7** | 10.08201 | 11.71245 | 11.59063 | 11.10817 | 13.00725 | 11.61801 | 12.34134 | 12.41375 | 1.221768 | 11.12332 | 12.34509 | 0.042405065 | 0.107426164 |  |
| **NRGN** | 10.15541 | 10.12837 | 10.13783 | 9.362246 | 10.8425 | 10.70477 | 10.44622 | 10.222 | 0.607907 | 9.945965 | 10.55387 | 0.047813951 | 0.1172213 |  |
| **Vimentin** | 10.03103 | 13.44924 | 14.19766 | 13.38985 | 16.62921 | 14.25696 | 15.76908 | 15.48654 | 2.768503 | 12.76695 | 15.53545 | 0.050968591 | 0.121050404 |  |
| **P62** | 7.759307 | 10.01145 | 9.940321 | 9.560926 | 11.08002 | 10.98231 | 10.65598 | 10.94245 | 1.597188 | 9.318 | 10.91519 | 0.054673107 | 0.124657622 |  |
| **C4B** | 4.247257 | 5.716743 | 6.146731 | 5.90437 | 7.876224 | 5.827314 | 7.139258 | 6.876224 | 1.42598 | 5.503775 | 6.929755 | 0.055767883 | 0.124657622 |  |
| **Ms IgG2a** | 4.093095 | 3.845168 | 4.237485 | 4.43013 | 4.179788 | 4.686748 | 4.838751 | 5.101786 | 0.550298 | 4.15147 | 4.701768 | 0.061210356 | 0.130757081 |  |
| **ATG5** | 7.446472 | 8.692783 | 8.375699 | 8.101336 | 9.459989 | 8.510321 | 8.702966 | 9.0459 | 0.775721 | 8.154073 | 8.929794 | 0.063469257 | 0.130757081 |  |
| **Ki-67** | 6.021021 | 7.076875 | 6.943019 | 6.554454 | 7.281964 | 7.031268 | 7.227857 | 7.647481 | 0.6483 | 6.648842 | 7.297142 | 0.065226434 | 0.130757081 |  |
| **BACE1** | 4.68581 | 6.074852 | 6.752924 | 6.359581 | 7.11998 | 6.741469 | 7.419541 | 7.463935 | 1.217939 | 5.968292 | 7.186231 | 0.067021919 | 0.130757081 |  |
| **Phospho-Tau (S404)** | 11.76851 | 12.77317 | 11.81736 | 12.87333 | 13.67097 | 12.30072 | 13.69061 | 13.63036 | 1.015075 | 12.30809 | 13.32317 | 0.067099028 | 0.130757081 |  |
| **CSF1R** | 8.228423 | 8.3439 | 8.400669 | 8.158817 | 9.628866 | 8.433374 | 8.996055 | 8.673425 | 0.649977 | 8.282952 | 8.93293 | 0.084306246 | 0.160181868 |  |
| **Amyloid Precursor Protein** | 10.31667 | 11.45659 | 11.84317 | 10.97689 | 12.1011 | 11.73094 | 11.85137 | 12.14012 | 0.807553 | 11.14833 | 11.95588 | 0.087005823 | 0.161279086 |  |
| **EMP1** | 9.503773 | 9.38039 | 8.799103 | 8.346881 | 10.38102 | 10.21224 | 9.284977 | 9.315214 | 0.790825 | 9.007537 | 9.798362 | 0.092480092 | 0.165860624 |  |
| **Phospho-Alpha-synuclein (S129)** | 3.598201 | 5.314408 | 4.598201 | 4.491286 | 4.87696 | 5.70985 | 5.162362 | 5.631847 | 0.844731 | 4.500524 | 5.345255 | 0.093842195 | 0.165860624 |  |
| **S6** | 12.45415 | 13.56745 | 14.06322 | 13.71522 | 14.61052 | 14.32918 | 13.62432 | 14.58638 | 0.83759 | 13.45001 | 14.2876 | 0.098629032 | 0.169130376 |  |
| **Tau** | 10.85805 | 13.72515 | 12.57468 | 13.06027 | 14.67323 | 13.28679 | 13.96923 | 13.95393 | 1.416256 | 12.55454 | 13.97079 | 0.100142986 | 0.169130376 |  |
| **S100B** | 14.72917 | 15.52043 | 14.28022 | 14.54474 | 16.33737 | 16.01211 | 15.1264 | 14.93032 | 0.83291 | 14.76864 | 15.60155 | 0.104972073 | 0.173432121 |  |
| **Neurofilament light** | 9.922364 | 10.99884 | 11.2616 | 10.06992 | 11.95219 | 10.77174 | 11.586 | 11.1189 | 0.794025 | 10.56318 | 11.35721 | 0.111893259 | 0.180933781 |  |
| **Calbindin** | 7.344335 | 11.20905 | 10.27376 | 10.15789 | 12.86275 | 10.32061 | 11.34049 | 11.67093 | 1.802436 | 9.74626 | 11.5487 | 0.126339433 | 0.200037435 |  |
| **PSEN1** | 7.622579 | 9.593724 | 9.564281 | 8.969992 | 9.539309 | 9.241681 | 10.60622 | 10.35454 | 0.997794 | 8.937644 | 9.935438 | 0.132906675 | 0.204798477 |  |
| **Tyrosine Hydroxylase** | 7.063233 | 8.621861 | 8.22193 | 7.928522 | 8.740262 | 8.446417 | 8.795891 | 8.506005 | 0.663257 | 7.958886 | 8.622144 | 0.136521939 | 0.204798477 |  |
| **Histone H3** | 12.32759 | 12.28659 | 13.00713 | 12.55002 | 13.26672 | 13.97926 | 12.40871 | 13.13054 | 0.653472 | 12.54283 | 13.19631 | 0.137430557 | 0.204798477 |  |
| **MERTK** | 6.827338 | 8.870265 | 9.800463 | 8.594012 | 10.63616 | 8.941749 | 9.552538 | 9.896633 | 1.233752 | 8.523019 | 9.756771 | 0.147929879 | 0.216205208 |  |
| **Amyloid-Beta 1-40** | 5.666301 | 6.059965 | 5.694316 | 5.971156 | 7.635334 | 7.752466 | 6.071745 | 5.765642 | 0.958362 | 5.847934 | 6.806296 | 0.159798146 | 0.226311377 |  |
| **Phospho-Tau (S199)** | 4.384163 | 6.957052 | 5.488499 | 6.016431 | 6.696525 | 6.840915 | 6.746278 | 6.53606 | 0.993408 | 5.711536 | 6.704944 | 0.160800189 | 0.226311377 |  |
| **CLEC7A** | 5.236617 | 5.763864 | 5.849594 | 5.236617 | 7.000567 | 5.708387 | 6.116045 | 5.553108 | 0.572853 | 5.521673 | 6.094527 | 0.183564126 | 0.253652247 |  |
| **TFEB** | 9.545181 | 9.287595 | 9.7003 | 9.117087 | 10.22906 | 9.85597 | 9.308347 | 9.652223 | 0.348858 | 9.412541 | 9.761399 | 0.190706528 | 0.258816002 |  |
| **Tdp-43** | 7.032902 | 9.914106 | 9.610775 | 9.283061 | 10.09247 | 9.321375 | 10.71183 | 9.832044 | 1.02922 | 8.960211 | 9.989431 | 0.22197997 | 0.291445068 |  |
| **IDE** | 5.92784 | 7.278747 | 7.5875 | 6.677297 | 7.809685 | 7.062072 | 7.237922 | 7.676419 | 0.578678 | 6.867846 | 7.446525 | 0.222418604 | 0.291445068 |  |
| **MAP2** | 14.11668 | 16.61554 | 16.62707 | 16.46134 | 16.26806 | 15.95557 | 17.85845 | 17.71301 | 0.993612 | 15.95516 | 16.94877 | 0.254516618 | 0.327851914 |  |
| **LRRK2** | 4.935664 | 6.950019 | 5.992248 | 5.501261 | 6.643721 | 6.362701 | 6.331337 | 6.408505 | 0.591768 | 5.844798 | 6.436566 | 0.260565251 | 0.330049319 |  |
| **CD9** | 13.31501 | 13.89897 | 13.55473 | 12.33115 | 14.78496 | 14.27149 | 12.93688 | 13.53645 | 0.607478 | 13.27497 | 13.88244 | 0.294768995 | 0.367253174 |  |
| **CTSD** | 13.20944 | 13.15583 | 12.92822 | 12.52426 | 13.26736 | 13.0955 | 13.0307 | 13.2094 | 0.196304 | 12.95444 | 13.15074 | 0.30428909 | 0.371545688 |  |
| **ATG12** | 6.315851 | 6.456332 | 7.644322 | 7.867646 | 7.383069 | 6.759217 | 7.937004 | 8.907964 | 0.675776 | 7.071038 | 7.746814 | 0.30799182 | 0.371545688 |  |
| **Park5** | 14.30014 | 15.92897 | 15.41291 | 15.6211 | 15.27661 | 16.12583 | 15.91051 | 15.75507 | 0.451224 | 15.31578 | 15.767 | 0.314157708 | 0.373062278 |  |
| **VPS35** | 7.536936 | 9.490807 | 9.342849 | 8.84874 | 8.722125 | 9.372379 | 9.484907 | 9.785819 | 0.536474 | 8.804833 | 9.341307 | 0.336172543 | 0.393063281 |  |
| **BAG3** | 6.136646 | 7.787799 | 6.630755 | 6.462632 | 8.099703 | 6.529387 | 7.726579 | 6.74552 | 0.520839 | 6.754458 | 7.275297 | 0.357044378 | 0.411142011 |  |
| **FUS** | 6.654823 | 8.801289 | 8.809151 | 8.147709 | 8.924373 | 8.420908 | 8.433152 | 8.775258 | 0.53518 | 8.103243 | 8.638423 | 0.373517877 | 0.42369192 |  |
| **PINK1** | 10.7553 | 8.875504 | 9.731184 | 8.523939 | 10.10569 | 11.10012 | 9.004966 | 10.16692 | 0.622945 | 9.471481 | 10.09443 | 0.380099206 | 0.42481676 |  |
| **HSC70** | 13.67551 | 10.17009 | 10.4614 | 8.663653 | 11.77365 | 13.93318 | 10.87297 | 11.12205 | 1.182798 | 10.74266 | 11.92546 | 0.390417723 | 0.429090865 |  |
| **NeuN** | 7.442052 | 11.40441 | 12.28768 | 11.37513 | 11.92696 | 10.38114 | 12.26571 | 12.37758 | 1.110529 | 10.62732 | 11.73785 | 0.39819588 | 0.429090865 |  |
| **P2ry12** | 6.438358 | 8.18483 | 9.130519 | 9.165153 | 9.224487 | 8.170937 | 8.584458 | 9.549915 | 0.652734 | 8.229715 | 8.882449 | 0.406291403 | 0.429090865 |  |
| **ApoA-I** | 9.274016 | 9.206011 | 10.93447 | 9.0426 | 9.537963 | 10.33751 | 9.46683 | 11.32965 | 0.553714 | 9.614273 | 10.16799 | 0.406507135 | 0.429090865 |  |
| **Synaptophysin** | 6.055339 | 9.606749 | 10.63823 | 9.860916 | 11.01557 | 7.716916 | 10.57379 | 11.10158 | 1.061657 | 9.040308 | 10.10196 | 0.44613147 | 0.46446564 |  |
| **GFAP** | 12.91087 | 13.52955 | 12.4029 | 12.65523 | 14.2565 | 11.99754 | 13.2244 | 13.65274 | 0.408159 | 12.87464 | 13.2828 | 0.484430179 | 0.497522886 |  |
| **P2RX7** | 8.981673 | 9.297596 | 10.78719 | 10.20804 | 11.03314 | 7.230443 | 12.04514 | 12.31215 | 0.836595 | 9.818624 | 10.65522 | 0.540969999 | 0.548182932 |  |
| **IBA1** | 8.68386 | 9.224609 | 9.40095 | 10.17487 | 9.762552 | 8.199063 | 9.736631 | 9.273063 | -0.12825 | 9.371073 | 9.242827 | 0.797820262 | 0.797820262 |  |

**Table S5. p-value sheet of microglia (Iba1)**

|  | **Ctl_Iba1_1** | **Ctl_Iba1_2** | **Ctl_Iba1_3** | **Ctl_Iba1_4** | **AD_Iba1_5** | **AD_Iba1_6** | **AD_Iba1_7** | **AD_Iba1_8** | **log Fold Change** | **mean_Ctl** | **mean_AD** | **t_test_p_val** | **p.adj** |
| --- | --- | --- | --- | --- | --- | --- | --- | --- | --- | --- | --- | --- | --- |
| **Neprilysin** | 11.785 | 11.56988 | 11.93903 | 12.55592 | 13.6615 | 14.51431 | 14.5808 | 14.0309 | 2.234419 | 11.96246 | 14.19688 | 0.000318683 | 0.02422 |
| **Amyloid-Beta 1-42** | 10.00795 | 10.12798 | 9.138051 | 9.91499 | 10.87149 | 10.78598 | 11.60448 | 11.91552 | 1.497123 | 9.797244 | 11.29437 | 0.006216381 | 0.127718 |
| **Phospho-Tau (S214)** | 7.957607 | 5.563692 | 5.137428 | 5.629281 | 9.968624 | 10.46802 | 9.797382 | 9.540658 | 3.87167 | 6.072002 | 9.943672 | 0.006244928 | 0.127718 |
| **Phospho-Tau (S396)** | 5.507395 | 8.349987 | 8.479381 | 9.021274 | 11.65503 | 11.29048 | 12.59688 | 12.91318 | 4.274384 | 7.839509 | 12.11389 | 0.00672198 | 0.127718 |
| **Ubiquitin** | 9.983318 | 9.440298 | 9.085972 | 9.402605 | 10.23735 | 9.916327 | 10.40067 | 10.09245 | 0.683652 | 9.478048 | 10.1617 | 0.026002349 | 0.269943 |
| **Alpha-synuclein** | 14.6053 | 15.15276 | 14.01752 | 14.28122 | 15.65898 | 15.45986 | 15.41915 | 15.17885 | 0.915009 | 14.5142 | 15.42921 | 0.02606468 | 0.269943 |
| **GAPDH** | 15.30375 | 15.38412 | 14.61179 | 14.5961 | 16.37925 | 15.56877 | 15.72529 | 15.57615 | 0.838428 | 14.97394 | 15.81237 | 0.027280299 | 0.269943 |
| **GBA** | 8.351038 | 7.956506 | 7.124706 | 7.312165 | 8.68884 | 8.680643 | 8.929219 | 8.817773 | 1.093015 | 7.686104 | 8.779119 | 0.028415006 | 0.269943 |
| **CD11c** | 9.927328 | 9.523813 | 10.6251 | 10.33988 | 10.4809 | 10.94883 | 11.06839 | 11.15595 | 0.809488 | 10.10403 | 10.91352 | 0.035449025 | 0.270479 |
| **S6** | 11.56 | 11.7759 | 11.85783 | 12.34794 | 12.45289 | 12.20279 | 12.34676 | 12.83745 | 0.574555 | 11.88542 | 12.45997 | 0.038281011 | 0.270479 |
| **GPNMB** | 6.33782 | 5.939271 | 6.094549 | 5.872157 | 7.375257 | 6.214792 | 7.864295 | 7.323726 | 1.133568 | 6.060949 | 7.194518 | 0.04228556 | 0.270479 |
| **LAMP2A** | 6.538429 | 6.35888 | 5.738728 | 6.475694 | 6.616643 | 6.715207 | 7.03168 | 7.495081 | 0.68672 | 6.277933 | 6.964653 | 0.043974044 | 0.270479 |
| **CD11b** | 9.333103 | 9.864716 | 9.605629 | 9.492941 | 10.62261 | 9.671377 | 10.54455 | 10.14098 | 0.670779 | 9.574098 | 10.24488 | 0.046266178 | 0.270479 |
| **Amyloid Precursor Protein** | 12.33813 | 11.55435 | 11.55482 | 11.28131 | 12.02817 | 12.54129 | 12.50352 | 12.29474 | 0.659778 | 11.68215 | 12.34193 | 0.055261327 | 0.28577 |
| **Vimentin** | 11.14488 | 13.74217 | 13.91833 | 13.59253 | 15.26302 | 13.71175 | 15.86182 | 15.32626 | 1.941235 | 13.09948 | 15.04071 | 0.056401888 | 0.28577 |
| **CD68** | 8.993322 | 9.321945 | 8.726256 | 9.439886 | 10.05054 | 9.182346 | 10.35991 | 9.716512 | 0.706973 | 9.120353 | 9.827325 | 0.063519402 | 0.301717 |
| **BACE1** | 5.817054 | 6.68581 | 6.522311 | 6.752924 | 6.602762 | 7.024403 | 7.806122 | 7.285789 | 0.735244 | 6.444525 | 7.179769 | 0.069107506 | 0.308951 |
| **Amyloid-Beta 1-40** | 6.608586 | 6.260662 | 4.948072 | 5.517438 | 9.144153 | 10.999 | 6.778698 | 6.765642 | 2.588184 | 5.83369 | 8.421873 | 0.080335103 | 0.329959 |
| **Park7** | 11.3559 | 12.02342 | 11.54683 | 11.71968 | 12.21535 | 11.59849 | 12.57821 | 12.48749 | 0.558426 | 11.66146 | 12.21988 | 0.085537764 | 0.329959 |
| **C4B** | 5.612907 | 6.176174 | 6.116673 | 6.956209 | 8.833519 | 6.273114 | 7.56179 | 7.36165 | 1.292028 | 6.215491 | 7.507518 | 0.086831446 | 0.329959 |
| **CD40** | 7.387253 | 7.227676 | 6.787104 | 7.667805 | 7.459362 | 7.532426 | 8.253272 | 7.899935 | 0.518789 | 7.267459 | 7.786249 | 0.092719689 | 0.335208 |
| **CTSD** | 12.53039 | 12.40975 | 11.6254 | 11.4467 | 12.85392 | 12.07861 | 12.84396 | 12.92928 | 0.67338 | 12.00306 | 12.67644 | 0.098284653 | 0.335208 |
| **IBA1** | 12.87428 | 14.2915 | 13.71881 | 14.75777 | 13.00585 | 11.61946 | 13.8005 | 12.47201 | -1.18614 | 13.91059 | 12.72445 | 0.101444629 | 0.335208 |
| **TMEM119** | 10.56477 | 11.86886 | 12.71805 | 12.48106 | 12.57163 | 12.43462 | 13.46976 | 13.42502 | 1.067073 | 11.90818 | 12.97526 | 0.115355529 | 0.365293 |
| **APOE** | 12.7449 | 11.26177 | 11.64191 | 10.74025 | 12.38082 | 12.58062 | 12.66366 | 12.37139 | 0.901916 | 11.59721 | 12.49912 | 0.122439423 | 0.372216 |
| **Calbindin** | 8.68785 | 11.19102 | 10.19253 | 10.6146 | 11.83905 | 10.21618 | 11.52835 | 11.56152 | 1.114774 | 10.1715 | 11.28628 | 0.142341416 | 0.399454 |
| **ADAM10** | 7.157752 | 6.42811 | 5.678918 | 5.824769 | 6.855171 | 7.112559 | 6.931986 | 6.749989 | 0.640039 | 6.272388 | 6.912426 | 0.152217717 | 0.399454 |
| **Phospho-Tdp-43 (S409/S410)** | 5.877268 | 6.110467 | 5.436696 | 5.253832 | 5.833046 | 5.97055 | 6.335547 | 6.055439 | 0.37908 | 5.669566 | 6.048645 | 0.15563839 | 0.399454 |
| **Phospho-Alpha-synuclein (S129)** | 5.183163 | 5.491286 | 4.434702 | 4.920129 | 3.87696 | 4.577399 | 4.831156 | 4.734941 | -0.50221 | 5.00732 | 4.505114 | 0.157378855 | 0.399454 |
| **CD163** | 6.060924 | 6.320987 | 7.502434 | 9.953495 | 9.941755 | 9.106097 | 11.09021 | 7.365617 | 1.91646 | 7.45946 | 9.37592 | 0.157679235 | 0.399454 |
| **Olig2** | 8.640546 | 8.269766 | 8.560857 | 8.55729 | 8.313613 | 8.797328 | 9.740574 | 9.372267 | 0.548831 | 8.507115 | 9.055945 | 0.178845721 | 0.43846 |
| **CD39** | 8.395188 | 7.343789 | 7.901079 | 7.633975 | 8.112746 | 8.748783 | 8.478202 | 7.777762 | 0.460865 | 7.818508 | 8.279373 | 0.185287435 | 0.440058 |
| **CD31** | 9.171046 | 8.95078 | 8.536734 | 8.893126 | 8.758411 | 9.130782 | 9.454077 | 9.467675 | 0.314815 | 8.887922 | 9.202737 | 0.192230121 | 0.442712 |
| **HLA-DR** | 9.129679 | 8.269857 | 7.514969 | 8.381967 | 8.487606 | 8.680251 | 9.011168 | 9.369084 | 0.562909 | 8.324118 | 8.887027 | 0.203384058 | 0.454623 |
| **Tau** | 12.09252 | 14.19721 | 12.61092 | 13.5273 | 13.97563 | 13.27257 | 14.181 | 13.97083 | 0.743023 | 13.10698 | 13.85001 | 0.217706725 | 0.472735 |
| **Phospho-Tau (T231)** | 7.297336 | 8.279073 | 7.87927 | 7.897342 | 7.955386 | 8.358451 | 8.376373 | 7.894504 | 0.307923 | 7.838255 | 8.146179 | 0.254544712 | 0.535834 |
| **Rb IgG** | 7.42347 | 7.246277 | 6.669488 | 7.196236 | 6.920845 | 7.282181 | 7.898477 | 7.904101 | 0.367533 | 7.133868 | 7.501401 | 0.260866588 | 0.535834 |
| **P62** | 7.902265 | 9.942529 | 9.136277 | 9.450469 | 9.043473 | 9.395236 | 10.50584 | 10.08982 | 0.650707 | 9.107885 | 9.758592 | 0.281543686 | 0.563087 |
| **CD45** | 9.604418 | 10.51965 | 10.74263 | 11.92948 | 11.63174 | 10.49938 | 12.21602 | 11.15468 | 0.67641 | 10.69905 | 11.37546 | 0.306310998 | 0.586633 |
| **LC3B** | 10.94647 | 10.44816 | 9.817723 | 9.789091 | 10.20054 | 10.61852 | 11.00283 | 10.64284 | 0.365824 | 10.25036 | 10.61618 | 0.309132653 | 0.586633 |
| **CSF1R** | 10.10004 | 10.20205 | 9.62571 | 10.72908 | 10.14145 | 9.096339 | 10.36003 | 9.412495 | -0.41164 | 10.16422 | 9.752577 | 0.316473187 | 0.586633 |
| **Ms IgG1** | 7.468025 | 7.285821 | 6.658631 | 6.966203 | 6.417163 | 6.985824 | 7.111355 | 6.930114 | -0.23356 | 7.09467 | 6.861114 | 0.359509214 | 0.633543 |
| **MERTK** | 8.363057 | 9.991874 | 10.64434 | 9.909666 | 10.57333 | 9.253693 | 10.69743 | 10.76425 | 0.594941 | 9.727235 | 10.32218 | 0.364126398 | 0.633543 |
| **Park5** | 15.49959 | 16.17333 | 15.22495 | 15.72928 | 14.35023 | 15.38152 | 15.83226 | 15.56659 | -0.37414 | 15.65679 | 15.28265 | 0.371574857 | 0.633543 |
| **Tdp-43** | 6.404871 | 7.797188 | 7.06438 | 7.437292 | 7.072751 | 6.933924 | 9.035886 | 7.86681 | 0.55141 | 7.175933 | 7.727343 | 0.37512425 | 0.633543 |
| **Myelin basic protein** | 16.62027 | 14.26546 | 14.36994 | 14.41989 | 14.85297 | 15.85612 | 15.34947 | 15.93477 | 0.579443 | 14.91889 | 15.49834 | 0.40211506 | 0.664364 |
| **EMP1** | 10.89689 | 9.54704 | 8.915428 | 8.970252 | 10.0573 | 10.50519 | 9.940225 | 9.620374 | 0.448368 | 9.582403 | 10.03077 | 0.417974816 | 0.675874 |
| **Ki-67** | 7.459594 | 7.021021 | 6.431305 | 10.07573 | 6.887432 | 6.591447 | 7.503091 | 7.227857 | -0.69446 | 7.746914 | 7.052457 | 0.457307658 | 0.709818 |
| **LRRK2** | 6.314176 | 6.441555 | 5.57721 | 6.421091 | 6.14547 | 6.523982 | 6.823542 | 6.108944 | 0.211977 | 6.188508 | 6.400485 | 0.457645821 | 0.709818 |
| **CLEC7A** | 6.592098 | 6.397082 | 5.358608 | 5.506707 | 5.678639 | 5.821597 | 6.047873 | 5.485994 | -0.2051 | 5.963624 | 5.758526 | 0.571819073 | 0.867119 |
| **S100B** | 16.62045 | 16.14415 | 14.55205 | 15.45628 | 15.94604 | 16.49293 | 15.84768 | 15.64651 | 0.290057 | 15.69323 | 15.98329 | 0.581882635 | 0.867119 |
| **Histone H3** | 12.06278 | 11.52724 | 12.05553 | 12.20094 | 11.22845 | 12.67371 | 12.48383 | 12.26745 | 0.201739 | 11.96162 | 12.16336 | 0.598821063 | 0.8752 |
| **ATG5** | 7.880568 | 8.908691 | 8.149271 | 8.367617 | 8.262049 | 8.001624 | 8.740933 | 8.872891 | 0.142838 | 8.326537 | 8.469374 | 0.649297545 | 0.891278 |
| **CD9** | 13.9918 | 12.96816 | 12.92652 | 11.69227 | 13.72298 | 13.58942 | 12.24893 | 13.09717 | 0.269937 | 12.89469 | 13.16462 | 0.658002062 | 0.891278 |
| **NeuN** | 7.548967 | 8.675724 | 9.428112 | 8.939881 | 8.29094 | 7.841364 | 9.839774 | 9.842952 | 0.305587 | 8.648171 | 8.953758 | 0.658573864 | 0.891278 |
| **TFEB** | 10.18498 | 9.798434 | 9.166084 | 9.489533 | 9.670345 | 9.631817 | 10.21678 | 9.594342 | 0.118564 | 9.659757 | 9.778321 | 0.669521949 | 0.891278 |
| **IDE** | 6.92784 | 7.540415 | 7.533561 | 7.389171 | 6.710907 | 7.140075 | 7.456932 | 7.647035 | -0.10901 | 7.347747 | 7.238737 | 0.68018539 | 0.891278 |
| **PSEN1** | 8.901006 | 9.953052 | 8.958721 | 9.139293 | 8.650526 | 9.009213 | 10.19458 | 9.827672 | 0.18248 | 9.238018 | 9.420498 | 0.689330355 | 0.891278 |
| **MAP2** | 14.66374 | 16.19098 | 16.26439 | 16.42458 | 15.13118 | 15.20686 | 17.3314 | 17.07698 | 0.300685 | 15.88592 | 16.18661 | 0.691913336 | 0.891278 |
| **P2ry12** | 9.383681 | 12.71132 | 13.30821 | 13.03026 | 11.2584 | 11.0542 | 11.81915 | 13.0068 | -0.32373 | 12.10837 | 11.78464 | 0.764829677 | 0.90568 |
| **Phospho-Tau (S199)** | 6.505178 | 9.757463 | 8.581871 | 9.645317 | 8.111562 | 8.57788 | 9.89568 | 8.913483 | 0.252194 | 8.622457 | 8.874651 | 0.778436508 | 0.90568 |
| **ApoA-I** | 11.04989 | 10.16742 | 11.28075 | 11.63768 | 9.166869 | 11.13527 | 11.15399 | 11.90095 | -0.19467 | 11.03393 | 10.83927 | 0.782113813 | 0.90568 |
| **PINK1** | 10.46668 | 8.732657 | 8.447071 | 7.779012 | 9.184135 | 9.77959 | 8.152192 | 9.086103 | 0.194151 | 8.856354 | 9.050505 | 0.782148991 | 0.90568 |
| **Tyrosine Hydroxylase** | 8.023549 | 8.903754 | 8.085428 | 8.490797 | 7.872277 | 8.185119 | 9.096517 | 8.729589 | 0.094993 | 8.375882 | 8.470875 | 0.790898253 | 0.90568 |
| **GFAP** | 14.85275 | 14.31643 | 12.81574 | 13.80415 | 14.14528 | 12.66083 | 13.98096 | 14.378 | -0.156 | 13.94727 | 13.79127 | 0.797171718 | 0.90568 |
| **HSC70** | 14.79041 | 10.25097 | 10.15637 | 9.139981 | 10.64716 | 13.63633 | 10.81584 | 10.81228 | 0.393472 | 11.08443 | 11.4779 | 0.797739428 | 0.90568 |
| **Phospho-Tau (S404)** | 13.25141 | 13.43466 | 11.8812 | 13.3587 | 12.89284 | 12.18856 | 13.83082 | 13.56647 | 0.138179 | 12.9815 | 13.11967 | 0.79964589 | 0.90568 |
| **FUS** | 6.986028 | 8.110017 | 7.648993 | 7.607517 | 7.531332 | 7.508371 | 7.768832 | 7.794367 | 0.062586 | 7.588139 | 7.650725 | 0.810572178 | 0.90568 |
| **Synaptophysin** | 7.39703 | 10.29144 | 10.8511 | 10.60037 | 10.4481 | 7.525186 | 11.02726 | 11.24632 | 0.276732 | 9.784984 | 10.06172 | 0.822262439 | 0.90568 |
| **VPS35** | 8.832392 | 10.22256 | 9.54699 | 9.38269 | 8.186406 | 9.482937 | 10.00341 | 9.933022 | -0.09471 | 9.496157 | 9.401444 | 0.859375971 | 0.933037 |
| **ATG12** | 8.13719 | 7.123206 | 7.964943 | 8.830834 | 6.542725 | 7.095266 | 8.626141 | 9.379226 | -0.1032 | 8.014043 | 7.91084 | 0.895885014 | 0.947354 |
| **BAG3** | 7.308827 | 8.470715 | 6.768259 | 7.701144 | 7.854226 | 6.758011 | 8.49622 | 7.41573 | 0.068811 | 7.562236 | 7.631047 | 0.897492818 | 0.947354 |
| **Neurofilament light** | 11.01173 | 11.32755 | 10.99235 | 10.54828 | 11.02729 | 10.43405 | 11.586 | 10.95416 | 0.030396 | 10.96998 | 11.00037 | 0.918987395 | 0.956754 |
| **NRGN** | 11.16122 | 10.40792 | 9.953249 | 9.668349 | 9.923957 | 10.40591 | 10.64716 | 10.33363 | 0.02998 | 10.29768 | 10.32767 | 0.937198106 | 0.962528 |
| **Ms IgG2a** | 4.799364 | 4.652523 | 3.932631 | 4.167096 | 4.101786 | 4.179788 | 5.019323 | 4.179788 | -0.01773 | 4.387903 | 4.370171 | 0.954397954 | 0.966001 |
| **P2RX7** | 10.81989 | 10.30702 | 11.30574 | 11.2685 | 10.75165 | 7.797213 | 12.8136 | 12.55562 | 0.054232 | 10.92529 | 10.97952 | 0.966000508 | 0.966001 |

**Table S6. p-value sheet of astrocytes (GFAP)**

|  | **Ctl_GFAP_1** | **Ctl_GFAP_2** | **Ctl_GFAP_3** | **Ctl_GFAP_4** | **AD_GFAP_5** | **AD_GFAP_6** | **AD_GFAP_7** | **AD_GFAP_8** | **log Fold Change** | **mean_Ctl** | **mean_AD** | **t_test_p_val** | **p.adj** |
| --- | --- | --- | --- | --- | --- | --- | --- | --- | --- | --- | --- | --- | --- |
| **Phospho-Tau (S214)** | 6.613161 | 3.459356 | 6.512467 | 5.692016 | 11.29266 | 10.78846 | 8.832524 | 8.450536 | 4.271795 | 5.56925 | 9.841045 | 0.005692492 | 0.278285 |
| **Amyloid-Beta 1-42** | 8.386295 | 8.518568 | 9.993608 | 9.838028 | 11.51917 | 11.36591 | 11.94751 | 11.05952 | 2.288905 | 9.184125 | 11.47303 | 0.007323297 | 0.278285 |
| **Phospho-Tau (S396)** | 5.534876 | 6.521201 | 9.488194 | 9.122105 | 13.04933 | 11.50231 | 11.6555 | 11.86551 | 4.351567 | 7.666594 | 12.01816 | 0.015255854 | 0.386482 |
| **GBA** | 6.287712 | 6.826738 | 7.9092 | 7.783669 | 8.904796 | 8.643168 | 8.400241 | 8.323348 | 1.366058 | 7.20183 | 8.567888 | 0.033123181 | 0.504094 |
| **Neprilysin** | 10.20372 | 9.926559 | 13.04107 | 12.61694 | 14.92685 | 14.74274 | 13.65657 | 13.51671 | 2.763645 | 11.44707 | 14.21072 | 0.033164054 | 0.504094 |
| **Alpha-synuclein** | 12.94489 | 13.36447 | 14.92634 | 14.34306 | 16.46655 | 15.60835 | 14.39381 | 14.94786 | 1.459452 | 13.89469 | 15.35414 | 0.061231099 | 0.626549 |
| **ADAM10** | 5.409732 | 4.824769 | 6.768186 | 5.647891 | 7.636531 | 7.238875 | 6.064624 | 6.636531 | 1.231496 | 5.662644 | 6.89414 | 0.061506792 | 0.626549 |
| **Ubiquitin** | 8.328605 | 8.015582 | 9.940121 | 9.125071 | 10.90799 | 10.32417 | 9.365699 | 9.649966 | 1.209613 | 8.852345 | 10.06196 | 0.073477653 | 0.626549 |
| **CD11c** | 6.49734 | 6.15749 | 8.723544 | 8.179858 | 9.348202 | 9.018609 | 8.812427 | 9.053125 | 1.668532 | 7.389558 | 9.058091 | 0.07419656 | 0.626549 |
| **GAPDH** | 13.74531 | 13.88952 | 15.51206 | 15.11542 | 16.97767 | 15.71537 | 15.20359 | 15.32082 | 1.238787 | 14.56558 | 15.80437 | 0.084464958 | 0.641899 |
| **APOE** | 10.86953 | 9.740251 | 12.62873 | 10.9497 | 13.15573 | 12.81622 | 11.68071 | 12.05138 | 1.378957 | 11.04706 | 12.42601 | 0.103085698 | 0.641899 |
| **Vimentin** | 10.04925 | 12.34504 | 14.94336 | 13.67427 | 16.29089 | 13.98854 | 14.90674 | 14.90433 | 2.269649 | 12.75298 | 15.02263 | 0.116164792 | 0.641899 |
| **TMEM119** | 7.799236 | 8.130113 | 10.47229 | 9.892345 | 10.90318 | 10.57393 | 10.07982 | 10.37903 | 1.410495 | 9.073496 | 10.48399 | 0.117427353 | 0.641899 |
| **LAMP2A** | 4.568803 | 5.193294 | 6.656266 | 6.80402 | 7.19674 | 6.929801 | 6.43023 | 7.053375 | 1.096941 | 5.805596 | 6.902536 | 0.13780364 | 0.641899 |
| **Amyloid-Beta 1-40** | 5.03827 | 4.453307 | 6.368915 | 5.666301 | 8.909094 | 12.47187 | 6.00665 | 6.028676 | 2.972374 | 5.381698 | 8.354073 | 0.146098149 | 0.641899 |
| **Calbindin** | 7.365922 | 9.567246 | 11.0581 | 10.58882 | 12.30992 | 10.57566 | 10.47713 | 11.37199 | 1.538651 | 9.645023 | 11.18367 | 0.163422188 | 0.641899 |
| **GPNMB** | 5.003401 | 4.801767 | 7.261199 | 6.312729 | 7.722276 | 5.968631 | 6.98531 | 7.157077 | 1.113549 | 5.844774 | 6.958324 | 0.164023659 | 0.641899 |
| **CD40** | 6.167376 | 5.787104 | 7.372066 | 7.636372 | 8.019124 | 7.311517 | 7.225999 | 7.716138 | 0.827465 | 6.740729 | 7.568194 | 0.165004837 | 0.641899 |
| **BACE1** | 4.380955 | 4.817054 | 7.54142 | 6.68581 | 7.174428 | 7.381473 | 6.765315 | 7.277522 | 1.293375 | 5.85631 | 7.149684 | 0.18347114 | 0.641899 |
| **LC3B** | 9.214533 | 8.730063 | 10.80507 | 10.07655 | 10.85998 | 10.73619 | 10.09453 | 10.29807 | 0.790639 | 9.706554 | 10.49719 | 0.186608857 | 0.641899 |
| **Olig2** | 7.078988 | 6.986542 | 9.020612 | 8.817942 | 8.900569 | 8.699932 | 8.868329 | 9.090722 | 0.913867 | 7.976021 | 8.889888 | 0.19282863 | 0.641899 |
| **Tau** | 10.6416 | 12.57604 | 13.57997 | 13.64497 | 14.74642 | 13.4745 | 13.21797 | 13.73669 | 1.183249 | 12.61065 | 13.7939 | 0.197214446 | 0.641899 |
| **CD11b** | 7.748141 | 7.482247 | 9.395991 | 9.098358 | 9.727411 | 9.168706 | 8.756557 | 9.222619 | 0.787639 | 8.431184 | 9.218823 | 0.202752281 | 0.641899 |
| **Rb IgG** | 6.21311 | 5.809213 | 7.752629 | 7.45927 | 7.926383 | 7.39033 | 7.238327 | 7.686379 | 0.751799 | 6.808556 | 7.560355 | 0.211152913 | 0.641899 |
| **C4B** | 5.13178 | 4.939135 | 6.90437 | 7.429461 | 8.988924 | 6.11908 | 7.129204 | 6.935117 | 1.191895 | 6.101186 | 7.293081 | 0.220363953 | 0.641899 |
| **CD45** | 7.901035 | 7.855644 | 9.588651 | 9.853996 | 10.20926 | 9.500491 | 9.254639 | 9.491599 | 0.814166 | 8.799831 | 9.613997 | 0.230793911 | 0.641899 |
| **Amyloid Precursor Protein** | 9.793037 | 10.49384 | 12.37667 | 11.79064 | 12.15496 | 12.11331 | 11.56081 | 12.0512 | 0.856522 | 11.11355 | 11.97007 | 0.244064686 | 0.641899 |
| **Phospho-Tau (T231)** | 6.006821 | 6.778869 | 8.508015 | 7.72592 | 8.654909 | 8.279697 | 7.445914 | 7.863072 | 0.805992 | 7.254906 | 8.060898 | 0.249056341 | 0.641899 |
| **Ms IgG2a** | 4.015093 | 3.652523 | 5.015093 | 4.015093 | 4.789842 | 4.179788 | 4.575717 | 4.789842 | 0.409347 | 4.17445 | 4.583797 | 0.272680148 | 0.641899 |
| **Phospho-Tdp-43 (S409/S410)** | 4.851733 | 4.131841 | 6.088772 | 5.927021 | 6.418009 | 5.733511 | 5.174083 | 6.369099 | 0.673834 | 5.249842 | 5.923675 | 0.273123076 | 0.641899 |
| **EMP1** | 8.826376 | 7.994499 | 9.689008 | 8.962897 | 10.26962 | 10.14769 | 8.588896 | 9.017133 | 0.637639 | 8.868195 | 9.505834 | 0.285161989 | 0.641899 |
| **S100B** | 15.41483 | 15.04795 | 15.73632 | 16.52305 | 16.76459 | 16.75757 | 15.79233 | 15.51427 | 0.526655 | 15.68054 | 16.20719 | 0.288036859 | 0.641899 |
| **Park7** | 9.775839 | 10.52515 | 12.61022 | 12.18146 | 12.75476 | 11.66895 | 11.91481 | 12.26324 | 0.87727 | 11.27317 | 12.15044 | 0.289813723 | 0.641899 |
| **HLA-DR** | 7.312926 | 6.823722 | 8.644252 | 8.621592 | 8.764174 | 8.529917 | 7.921837 | 8.614925 | 0.60709 | 7.850623 | 8.457713 | 0.290994309 | 0.641899 |
| **Phospho-Tau (S404)** | 11.67916 | 11.68592 | 12.88923 | 13.44573 | 13.66613 | 12.28027 | 12.82607 | 13.39622 | 0.617163 | 12.42501 | 13.04217 | 0.301775127 | 0.641899 |
| **CD31** | 7.959677 | 7.583528 | 9.196358 | 9.373235 | 9.337461 | 9.331524 | 8.638502 | 9.024393 | 0.554771 | 8.528199 | 9.08297 | 0.310360684 | 0.641899 |
| **CD68** | 8.030854 | 7.454583 | 9.004463 | 9.080127 | 9.283307 | 8.688337 | 8.598571 | 8.893895 | 0.473521 | 8.392507 | 8.866027 | 0.326333212 | 0.641899 |
| **CTSD** | 9.974906 | 11.32973 | 12.09086 | 12.02433 | 12.23061 | 11.73691 | 11.9746 | 11.74744 | 0.567434 | 11.35496 | 11.92239 | 0.335272513 | 0.641899 |
| **Myelin basic protein** | 15.40975 | 12.81213 | 15.46589 | 14.68629 | 15.38021 | 15.8342 | 14.39824 | 15.73339 | 0.742994 | 14.59352 | 15.33651 | 0.34212202 | 0.641899 |
| **P62** | 6.697907 | 8.459747 | 10.02401 | 9.820603 | 9.64272 | 9.301564 | 9.73394 | 9.748873 | 0.856207 | 8.750567 | 9.606774 | 0.34695097 | 0.641899 |
| **PSEN1** | 7.072892 | 8.356685 | 9.981176 | 9.295285 | 9.387026 | 9.112494 | 9.340186 | 9.598404 | 0.683018 | 8.676509 | 9.359528 | 0.359176204 | 0.641899 |
| **S6** | 9.786812 | 10.1785 | 12.55834 | 12.11553 | 12.13915 | 11.84997 | 11.31124 | 12.28341 | 0.736148 | 11.1598 | 11.89594 | 0.3723936 | 0.641899 |
| **CD163** | 4.721074 | 4.579055 | 7.196808 | 8.442173 | 7.637421 | 6.890708 | 8.095527 | 6.217518 | 0.975516 | 6.234778 | 7.210293 | 0.398594366 | 0.641899 |
| **PINK1** | 8.632069 | 7.171058 | 9.680189 | 7.714882 | 9.837647 | 9.915826 | 7.380724 | 8.934726 | 0.717681 | 8.29955 | 9.01723 | 0.407731178 | 0.641899 |
| **GFAP** | 14.02623 | 14.2294 | 14.52587 | 15.43486 | 15.65469 | 14.09699 | 14.81497 | 15.18467 | 0.383742 | 14.55409 | 14.93783 | 0.429075693 | 0.641899 |
| **TFEB** | 8.252204 | 8.223253 | 9.770356 | 9.444252 | 9.998264 | 9.502919 | 8.873457 | 8.938041 | 0.405654 | 8.922516 | 9.32817 | 0.435481412 | 0.641899 |
| **CD9** | 12.34024 | 11.42812 | 13.84751 | 11.81471 | 14.3532 | 13.65262 | 11.35707 | 12.8362 | 0.692125 | 12.35765 | 13.04977 | 0.439614479 | 0.641899 |
| **NRGN** | 9.542225 | 8.703814 | 10.98505 | 9.706995 | 10.63762 | 10.50272 | 9.605948 | 9.978563 | 0.446693 | 9.73452 | 10.18121 | 0.440838148 | 0.641899 |
| **IBA1** | 8.981786 | 8.941077 | 10.81356 | 11.65046 | 10.05966 | 9.045283 | 9.679056 | 9.131568 | -0.61783 | 10.09672 | 9.478892 | 0.441441403 | 0.641899 |
| **Tyrosine Hydroxylase** | 6.636968 | 7.527739 | 9.163141 | 8.531786 | 8.591013 | 8.28083 | 8.190318 | 8.761375 | 0.490976 | 7.964908 | 8.455884 | 0.447763027 | 0.641899 |
| **Tdp-43** | 5.337757 | 6.484598 | 7.530402 | 7.461139 | 7.111922 | 6.370656 | 8.09247 | 7.281847 | 0.51075 | 6.703474 | 7.214224 | 0.448017538 | 0.641899 |
| **ATG5** | 6.646771 | 6.774526 | 9.313948 | 8.334827 | 9.075918 | 8.178051 | 7.628965 | 8.553632 | 0.591623 | 7.767518 | 8.359141 | 0.449426291 | 0.641899 |
| **FUS** | 5.829909 | 6.809151 | 8.26668 | 7.552375 | 8.332164 | 7.190295 | 7.161149 | 7.654964 | 0.470114 | 7.114529 | 7.584643 | 0.464402325 | 0.641899 |
| **MERTK** | 6.630538 | 7.851344 | 10.93628 | 9.62637 | 10.62403 | 8.819445 | 9.061048 | 9.842468 | 0.825615 | 8.761134 | 9.586749 | 0.468949342 | 0.641899 |
| **Neurofilament light** | 9.428375 | 9.748378 | 11.92748 | 10.62267 | 11.6821 | 10.57011 | 10.60875 | 10.79133 | 0.481344 | 10.43173 | 10.91307 | 0.476300169 | 0.641899 |
| **LRRK2** | 4.75124 | 5.099163 | 6.799603 | 6.149789 | 6.481754 | 6.090329 | 5.823542 | 5.952825 | 0.387164 | 5.699948 | 6.087112 | 0.481398275 | 0.641899 |
| **HSC70** | 12.98028 | 8.546117 | 11.03958 | 8.979218 | 11.22357 | 13.76466 | 9.901379 | 10.63351 | 0.994482 | 10.3863 | 11.38078 | 0.4814246 | 0.641899 |
| **IDE** | 5.677297 | 5.989241 | 8.422942 | 7.335016 | 7.814979 | 7.253604 | 6.641086 | 7.641086 | 0.481565 | 6.856124 | 7.337689 | 0.521212561 | 0.682968 |
| **MAP2** | 13.18707 | 14.56302 | 17.48421 | 16.52679 | 15.98771 | 15.2745 | 16.31284 | 16.94161 | 0.688895 | 15.44027 | 16.12916 | 0.541174677 | 0.697106 |
| **Histone H3** | 10.04021 | 9.905367 | 12.04301 | 12.30767 | 11.10696 | 11.95072 | 11.30316 | 11.57735 | 0.410482 | 11.07407 | 11.48455 | 0.574698332 | 0.727951 |
| **Ms IgG1** | 6.523981 | 6.248347 | 7.45163 | 7.484235 | 7.441276 | 6.963798 | 6.799411 | 7.236051 | 0.183086 | 6.927048 | 7.110134 | 0.625490731 | 0.779092 |
| **CSF1R** | 7.756523 | 7.611752 | 9.605728 | 8.911061 | 9.527648 | 8.552672 | 8.529589 | 8.378447 | 0.275823 | 8.471266 | 8.747089 | 0.635575274 | 0.779092 |
| **Synaptophysin** | 6.640301 | 8.664964 | 11.74762 | 10.75769 | 11.30081 | 7.942053 | 9.983952 | 11.09172 | 0.62699 | 9.452645 | 10.07963 | 0.665935652 | 0.787271 |
| **Ki-67** | 5.655737 | 5.982547 | 7.586875 | 7.624843 | 7.152404 | 6.87575 | 6.619736 | 7.190624 | 0.247128 | 6.712501 | 6.959629 | 0.673255632 | 0.787271 |
| **VPS35** | 7.443154 | 8.541972 | 10.51277 | 9.348609 | 8.883117 | 9.643194 | 8.958759 | 9.605673 | 0.311059 | 8.961627 | 9.272686 | 0.6733243 | 0.787271 |
| **NeuN** | 6.989539 | 7.256185 | 10.22773 | 8.925779 | 9.009169 | 7.498631 | 8.850866 | 9.526568 | 0.371501 | 8.349808 | 8.721309 | 0.689011923 | 0.793408 |
| **P2RX7** | 9.21988 | 8.333261 | 12.35718 | 11.15682 | 11.28303 | 7.969577 | 11.56802 | 12.30122 | 0.513676 | 10.26678 | 10.78046 | 0.711767563 | 0.807378 |
| **Phospho-Alpha-synuclein (S129)** | 3.878309 | 4.250278 | 5.572206 | 5.076248 | 5.162362 | 4.631847 | 4.046885 | 5.658319 | 0.180593 | 4.69426 | 4.874853 | 0.739428119 | 0.82642 |
| **ATG12** | 7.732936 | 5.500275 | 10.02384 | 8.925197 | 6.978227 | 6.850847 | 7.569947 | 9.296085 | -0.37178 | 8.045561 | 7.673777 | 0.753988536 | 0.83048 |
| **BAG3** | 5.741786 | 7.887766 | 8.014805 | 9.00649 | 8.949879 | 6.782673 | 8.641497 | 7.283734 | 0.251734 | 7.662712 | 7.914446 | 0.781167338 | 0.848125 |
| **Phospho-Tau (S199)** | 5.616823 | 8.85665 | 8.755722 | 10.27655 | 7.448597 | 8.564074 | 8.693357 | 7.683814 | -0.27898 | 8.376437 | 8.097461 | 0.801588176 | 0.858038 |
| **CD39** | 6.018119 | 4.960785 | 6.980151 | 6.274894 | 6.384419 | 6.872919 | 5.675882 | 5.675882 | 0.093788 | 6.058487 | 6.152276 | 0.8610351 | 0.90887 |
| **ApoA-I** | 9.431199 | 8.81614 | 12.57966 | 12.37594 | 9.633858 | 11.16767 | 10.01487 | 11.76513 | -0.15535 | 10.80073 | 10.64538 | 0.893447461 | 0.930164 |
| **CLEC7A** | 5.007136 | 5.149155 | 6.541472 | 6.103351 | 6.926567 | 5.22296 | 5.263602 | 5.263602 | -0.0311 | 5.700278 | 5.669183 | 0.957547209 | 0.978397 |
| **Park5** | 13.90653 | 14.46429 | 16.2288 | 15.81135 | 15.03076 | 15.39165 | 14.74402 | 15.31351 | 0.01724 | 15.10274 | 15.11998 | 0.977460768 | 0.978397 |
| **P2ry12** | 6.795619 | 7.814115 | 10.58558 | 10.05768 | 9.267641 | 8.389862 | 8.140305 | 9.344898 | -0.02757 | 8.81325 | 8.785676 | 0.978396968 | 0.978397 |
